# Transdifferentiated BLaER1 cells as a genetically tractable model to study the interaction of pathogenic fungi with macrophages

**DOI:** 10.1242/dmm.052865

**Published:** 2026-08-05

**Authors:** Johannes Sonnberger, Erik Böhm, Janik Adriana Tomás-Morales, Theresa Lange, Katrin Bagola, Lisa Denner, Lydia Kasper, Sascha Brunke, Ger van Zandbergen, Jakob L. Sprague, Bernhard Hube

**Affiliations:** ^1^ Department of Microbial Pathogenicity Mechanisms, Hans Knöll Institute, 07745 Jena, Germany; ^2^ Department of Medicine, Health and Medical University Erfurt, 99089 Erfurt, Germany; ^3^ Division of Immunology, Paul Ehrlich Institute Langen, 63225 Langen, Germany; ^4^ Institute of Novel and Emerging Infectious Diseases, Friedrich Loeffler Institute, Federal Research Institute for Animal Health, 17493 Greifswald-Insel Riems, Germany; ^5^ Institut for Immunology, University Medical Center, Johannes Gutenberg University, 55131 Mainz, Germany; ^6^ Research Center for Immunotherapy (FZI), University Medical Center, Johannes Gutenberg University, 55131 Mainz, Germany; ^7^ Institute of Experimental Biomedicine, Chair I, University Hospital Würzburg, 97080 Würzburg, Germany; ^8^ Rudolf Virchow Center for Integrative and Translational Bioimaging, Julius-Maximilians-Universität, 97080 Würzburg, Germany; ^9^ Institute of Microbiology, Friedrich Schiller University, 07743 Jena, Germany; ^10^ Cluster of Excellence, Balance of the Microverse, Friedrich Schiller University, 07745 Jena, Germany

**Keywords:** Innate immunity, Fungal pathogens, Human infection model

## Abstract

Macrophages are essential in the defense against fungal disease. Elucidating antimicrobial mechanisms of macrophages and pathogen activities to evade these phagocytes will help in understanding fungal infections. Existing experimental models, however, have a number of disadvantages. Human macrophage-like cell lines offer genetic tractability, but often display reduced antifungal activity and altered phenotypes compared to primary cells. In contrast, primary human monocyte-derived macrophages closely reflect physiological responses but their use is constrained by donor variability, limited availability and restricted genetic manipulability. Here, we establish transdifferentiated BLaER1 cells as a human-derived, highly potent and genetically tractable infection model to study fungal–host interactions. We show that BLaER1 cells display macrophage characteristics and rapidly phagocytose cells of the major fungal pathogens *Candida albicans*, *C. glabrata* and *C. auris*. BLaER1 cells form functional phagolysosomes and elicit pro-inflammatory immune responses. Using knockout BLaER1 cells, we demonstrate for the first time in a human macrophage model that the host factor gasdermin D and the fungal peptide toxin candidalysin have distinct roles in triggering pyroptosis and inducing lytic host cell death.

## Teaser

**Summary:** Transdifferentiated BLaER1 cells, a human-derived, genetically tractable macrophage infection model of fungal pathogens, closely resembles primary human macrophages in pro-inflammatory responses.

## INTRODUCTION

Fungal infections still represent an understudied risk to human health with invasive candidiasis being among the most prevalent forms (Denning, 2024a). Recognizing this, the WHO has categorized one of the most frequent fungal pathogens, *Candida albicans*, and the newly emerged *C. auris* (recently re-classified as *Candidozyma auris*) into a critical priority group (WHO, 2022). Furthermore, *C. glabrata* (recently re-classified as *Nakaseomyces glabratus*), the second most prevalent cause of candidiasis, was designated a high priority pathogen (WHO, 2022). While phylogenetically distinct, all three pathogens will be referred to as *Candida* spp. in this study for pragmatism and in line with clinical use in medical mycology (Denning, 2024b).

Macrophages are an important first-line defense against invasive fungal infections (Erwig and Gow, 2016). Thus, studying *Candida*–macrophage interactions is of central importance in understanding pathogenicity of these phylogenetically diverse fungi. All three pathogens share the ability to survive phagocytosis by macrophages and are able to subsequently escape from the host cell via different mechanisms (Lange et al., 2023; Sonnberger et al., 2024). *C. albicans* induces pro-inflammatory forms of cell death pathways, mostly pyroptosis (Uwamahoro et al., 2014; Wellington et al., 2014), but also necroptosis (Banoth et al., 2020). Furthermore, *C. albicans* can trigger macrophage cell death via depletion of glucose (Tucey et al., 2018) and by the fungal pore-forming peptide toxin candidalysin, which is associated with the secretion of pro-inflammatory cytokines (Kasper et al., 2018). Moreover, the fungus can escape from the phagolysosome via hypha formation (Ghosh et al., 2009; Westman et al., 2018). *C. auris* induces macrophage lysis (Miramón et al., 2023; Weerasinghe et al., 2023) due to increased glycolysis, followed by rapid proliferation (Weerasinghe et al., 2023). In contrast to *C. albicans*, *C. auris* does not induce a significant pro-inflammatory cytokine release (Wang et al., 2022). Similarly, *C. glabrata* does not elicit pro-inflammatory host responses, but can persist in macrophages for days and escape by replication, leading to bursting of the host cell (Lange et al., 2026; Seider et al., 2011).

Robust macrophage infection models are essential to dissect host–*Candida* interactions at the cellular and molecular level. Commonly used systems include murine macrophage cell lines (Ghosh et al., 2009; O'Meara et al., 2018; Silao et al., 2019; Vylkova and Lorenz, 2017) or primary mouse cells (Ding et al., 2021; Kasper et al., 2018; Olivier et al., 2022), which are genetically tractable but limited by species-specific differences, as laboratory mice are not natural hosts of *Candida* spp. (Mishra and Koh, 2021; Rosshart et al., 2019). Human macrophage-like cell lines offer genetic tractability but often display reduced antifungal activity and altered phenotypes compared to primary cells (Daigneault et al., 2010; Liu et al., 2019). In contrast, primary human monocyte-derived macrophages (hMDMs) most closely reflect physiological antifungal responses. While gene knockouts have been successfully created in hMDMs (Freund et al., 2020), genetic insertions are still challenging. Furthermore, their use is constrained by donor variability and limited availability (Ter Horst et al., 2016). These existing experimental models based on immortalized cell lines or primary macrophages have led to groundbreaking discoveries in medical mycology, but also have limitations and a number of disadvantages.

To overcome some challenges of existing models, we investigated the potential of transdifferentiated BLaER1 cells to model human macrophage–*Candida* interactions. BLaER1 cells originate from the B cell leukemia C/EBPαER clone 1 line derived from RCH-ACV cells, and they robustly transdifferentiate into macrophage-like cells upon induction with IL-3, β-estradiol and M-CSF (Rapino et al., 2013). Owing to their resemblance to hMDMs (Rapino et al., 2013; Volkmar et al., 2024) and the expression of key immune components, including canonical and non-canonical inflammasomes (Gaidt et al., 2016, 2018), BLaER1 cells have been successfully used to study infections with bacteria (Nunes et al., 2024), archaea (Vierbuchen et al., 2017), parasites (Volkmar et al., 2024) and viruses (Gaidt et al., 2021) and have been shown to phagocytose *C. albicans* (Rapino et al., 2013). Here, we describe transdifferentiated BLaER1 cells as a genetically tractable human-derived macrophage model to study pathogenicity and immune evasion of *C. albicans*, *C. glabrata* and *C. auris*. They recapitulate key aspects of macrophage functionality during interactions with *Candida* spp. BLaER1 cells possess high antifungal capacity and proved to be particularly useful to study the effect of intracellular filamentation and the filamentation-associated escape of *C. albicans* from macrophages.

By leveraging their genetic tractability, we dissect the distinct roles of filamentation, candidalysin secretion and gasdermin D in the induction of host cell death and IL-1β release for the first time in a human-derived macrophage infection model. We demonstrate that candidalysin and gasdermin D are the two key factors resulting in *C. albicans*-induced IL-1β release, which can be mediated by either factor independently. Thus, we are able to confirm the hypothesis that both factors contribute to *C. albicans*-induced host cell death in humans.

## RESULTS

### Transdifferentiated BLaER1 cells acquire a macrophage-like morphology and express antifungal pattern-recognition receptors

**Fig. 1.**
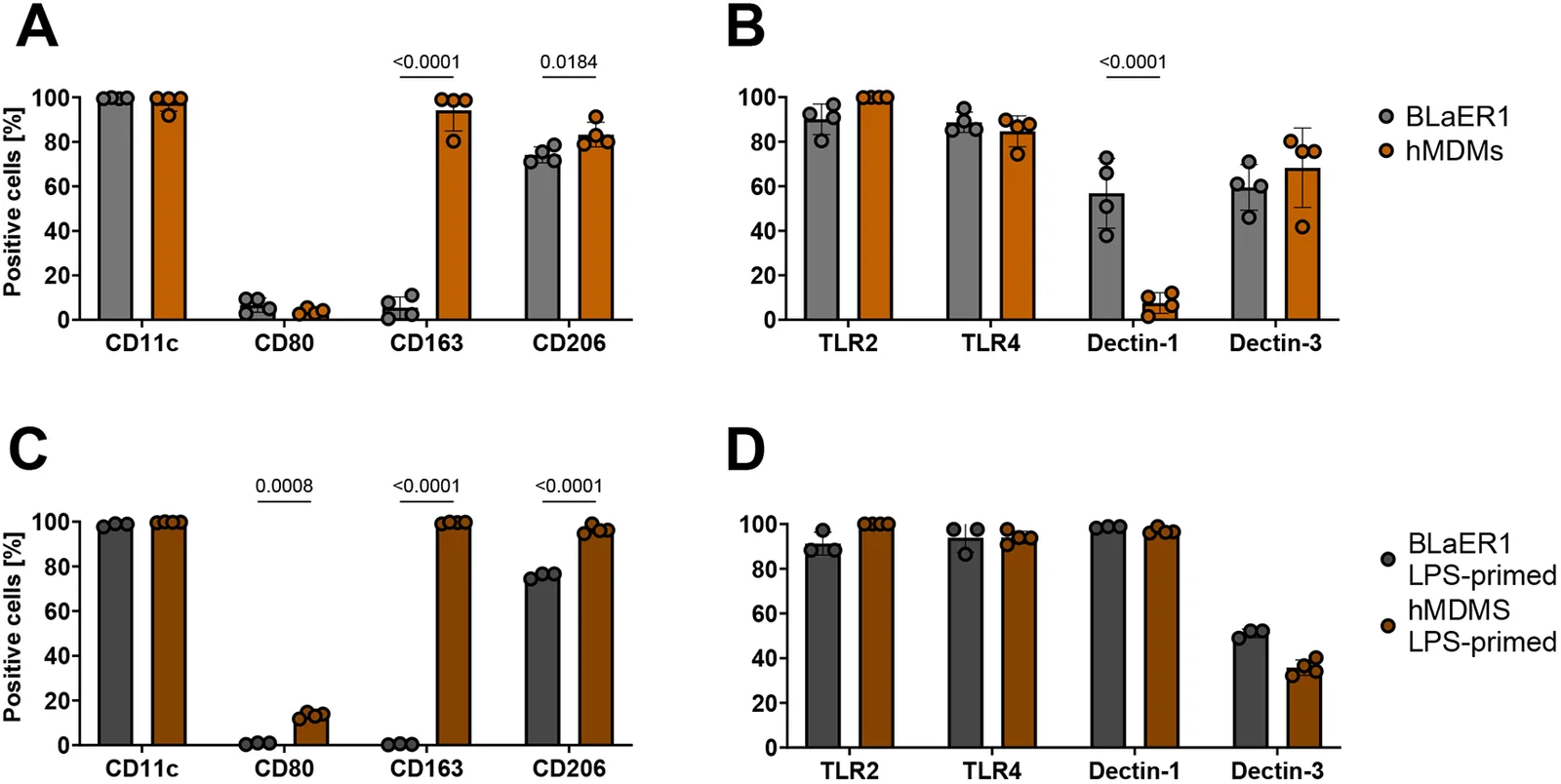
**Transdifferentiated and LPS-primed BLaER1 cells phenotypically resemble human macrophages.** (A-D) Expression of macrophage polarization markers (A) and selected PRRs (B) by transdifferentiated BLaER1 cells and M-CSF-differentiated hMDMs was analyzed by flow cytometry. Expression was additionally quantified after LPS priming for polarization markers (C) and PRRs (D). The number of positive cells was determined as percentage of CD14^+^ population. Data are displayed as mean±s.d. and were analyzed with a two-way ANOVA and Bonferroni's multiple comparisons test (BLaER1: *n*=3 and 4; hMDMs: 4 donors). Only *P*-values below 0.05 are indicated.

Macrophage-like BLaER1 cells can be generated by transdifferentiation with IL-3, β-estradiol and macrophage colony-stimulating factor (M-CSF) (Rapino et al., 2013). For further characterization and comparison of BLaER1 cells to M-CSF-differentiated hMDMs, the expression of polarization markers indicative of the activation state of macrophages (Wynn et al., 2013) was quantified in both cell types (Fig. 1A). Additionally, the expression of pattern recognition receptors (PRRs) for the detection of fungal pathogen-associated molecular patterns, dectin-1 (CLEC7A) (Brown et al., 2002) and dectin-3 (CLEC4D/MCL) (Zhu et al., 2013) was analyzed (Fig. 1B).

Efficient transdifferentiation of BLaER1 cells to adherent macrophage-like cells was monitored as described before (Rapino et al., 2013) (Fig. S1A-D). From here on, transdifferentiated BLaER1 cells will be referred to as BLaER1 cells.

M1 polarization markers include CD11c (ITGAX), CD80 as well as the PRRs TLR2 and TLR4 (Shapouri-Moghaddam et al., 2018). Characteristic M2 polarization markers include CD163 and CD206 (MRC1) (Murray et al., 2014). Both BLaER1 and hMDMs were mostly CD11c^+^ and CD80^−^ (Fig. 1A). While BLaER1 cells were generally CD163^−^, over 90% of hMDMs were CD163^+^ (Fig. 1A). A significantly higher proportion of hMDMs was also CD206^+^ (Fig. 1A). Both cell types were over 80% TLR2/4^+^ (Fig. 1B). Between 60% and 70% of BLaER1 and hMDMs were dectin-3^+^. Interestingly, significantly more BLaER1 cells than hMDMs were dectin-1^+^ (Fig. 1B).

Taken together, following transdifferentiation BLaER1 cells displayed more characteristics typical for M1 polarization (CD14^+^, CD80^−^, CD163^−^, TLR2/4^+^).

Lipopolysaccharide (LPS) treatment for 2 h is a well-established method for inflammasome priming of macrophages prior to infection studies (Kasper et al., 2018; Tucey et al., 2018). We repeated our analyses with LPS-primed BLaER1 cells and hMDMs. Following priming, the majority of BLaER1 cells became more elongated (Fig. S1A) and we detected fewer CD80^+^ BLaER1 cells, but more CD80^+^ hMDMs (Fig. 1C). In contrast, over 95% of both BLaER1 and hMDMs were dectin-1^+^ after LPS priming (Fig. 1D). The fraction of dectin-3^+^ cells decreased to a similar degree for hMDMs and BLaER1 cells.

The expression of CD11c, CD163 and TLR2/4 was unaffected by LPS priming (Fig. 1D). To better understand the reaction of these LPS-primed BLaER1 cells and hMDMs to infections, we turned to transcriptional profiling.

### LPS-primed BLaER1 cells closely recapitulate the transcriptional response of LPS-primed hMDMs to *C. albicans* infection

**Fig. 2.**
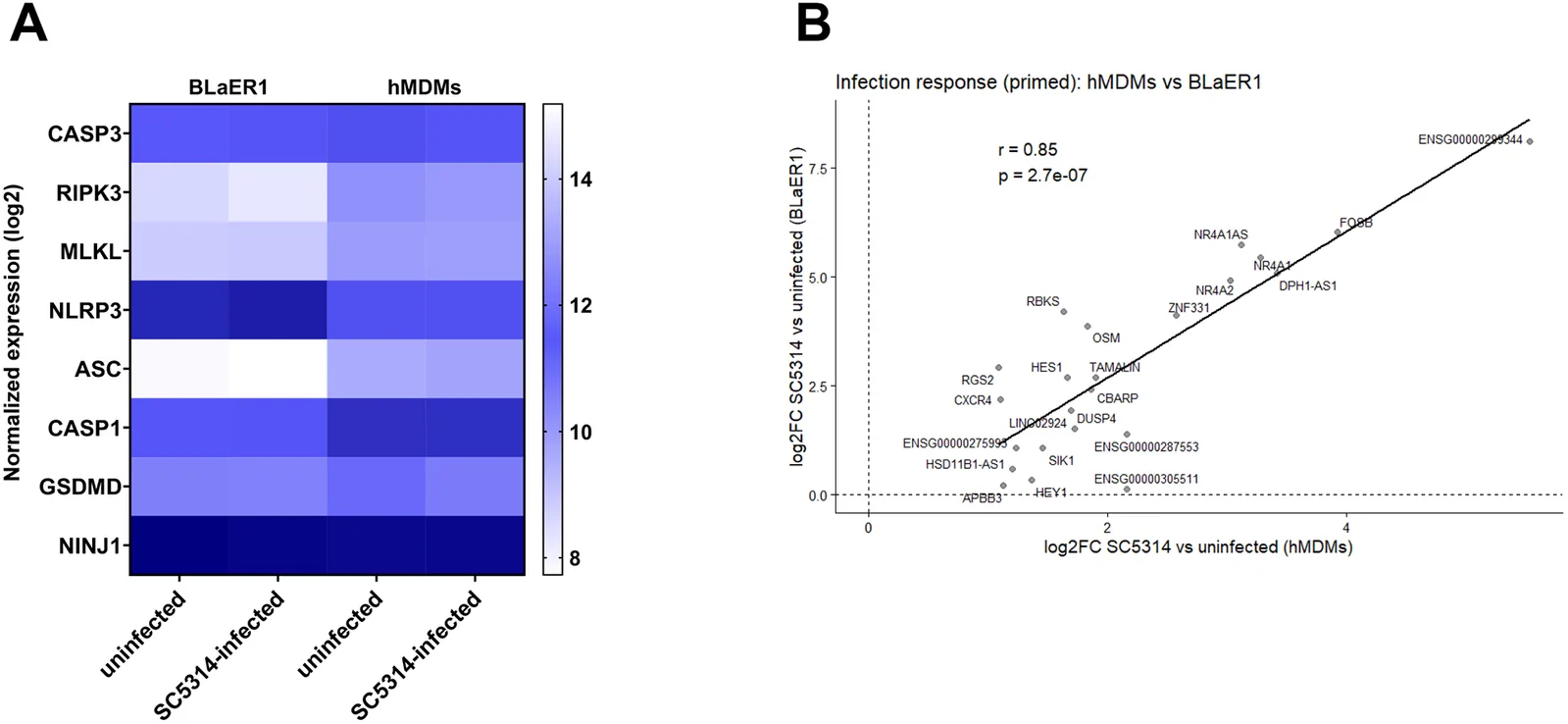
**LPS-primed BLaER1 cells and hMDMs respond similarly to *C. albicans* infection.** Gene expression in LPS-primed BLaER1 cells and LPS-primed hMDMs was analyzed by RNA sequencing of either uninfected samples or after 2 h of infection with *C. albicans* SC5314 WT. (A) Normalized log2-transformed expression of selected genes involved in the execution of programmed cell death pathways in uninfected and *C. albicans* SC5314 WT-infected, LPS-primed BLaER1 cells and LPS-primed hMDMs. (B) Log2-transformed fold change (log2FC) of DEGs (adjusted *P*<0.05) in response to SC5314 infection in LPS-primed hMDMs plotted against log2FC for the same genes after infection in LPS-primed BLaER1 cells. Correlation was quantified with Spearman's rank correlation coefficient (BLaER1: *n*=5; hMDMs: 5 donors).

During transdifferentiation, BLaER1 cells become transcriptionally more similar to hMDMs (Rapino et al., 2013). Expanding on that, we compared LPS-primed BLaER1 cells and hMDMs to each other and in their response to infection with *C. albicans* using RNA sequencing. In direct comparison, the global transcriptomes of uninfected, LPS-primed BLaER1 cells and hMDMs display profound differences, reflecting their different cellular histories (Table S1). Despite these differences, gene sets related to specific molecular functions can still be expressed to a similar degree. During infection, undergoing programmed cell death is an important trait of macrophages (Chow et al., 2016). Prominent examples (with selected marker genes) are: apoptosis (*CASP3*), necroptosis (*RIPK3*, *MLKL*), pyroptosis [*NLRP3*, *ASC* (*PYCARD*), *CASP1*, *GSDMD*] and a central downstream effector of lytic cell death (*NINJ1*). The median-ratio-normalized, log2-transformed expression pattern of these genes was highly similar among both LPS-primed cell types (Fig. 2A). Most components were expressed to a slightly higher degree in LPS-primed hMDMs, with the exception of *NLRP3*. *CASP3*, *GSDMD* and *NINJ1* were expressed at almost identical levels in both cell types. The relative expression levels of these genes within each cell type followed the same pattern. This shows that, for specific macrophage functions, such as programmed cell death, LPS-primed BLaER1 cells are similarly prepared. This can be required, for example, during fungal infections.

Accordingly, the selected genes showed no further transcriptional change in response to infection with *C. albicans* wild type (WT) in either BLaER1 cells or in hMDMs (Fig. 2A). Indeed, the general transcriptional response to 2 h of *C. albicans* infection was moderate in both LPS-primed cell types, with few statistically significantly differentially expressed genes (DEGs; Fig. 2B). The DEGs we found included genes encoding the transcription factors FOSB, NR4A1 and NR4A2, which have previously been linked to the response to *C. albicans* infection (Zhu et al., 2022). Additionally, CXCR4, which encodes a chemokine receptor involved in immune responses and the interaction with LPS (Triantafilou et al., 2001), was also upregulated during infection with *C. albicans* in both LPS-primed BLaER1 cells and hMDMs. Importantly, a comparison of the log2-fold change of the DEGs in response to *C. albicans* infection between LPS-primed BLaER1 cells and hMDMs showed that they were highly correlated (Fig. 2B; r=0.85), suggesting that the pattern of the transcriptional response of hMDMs to fungal infections is mirrored well in BLaER1 cells, despite their different backgrounds.

In summary, BLaER1 cells are equipped to recognize, phagocytose and respond to fungal pathogens similarly to primary human macrophages both before and after LPS priming. Next, we aimed to characterize different stages of this response.

### Phagocytosis of *C. albicans*, *C. glabrata* and *C. auris* by BLaER1 cells

**Fig. 3.**
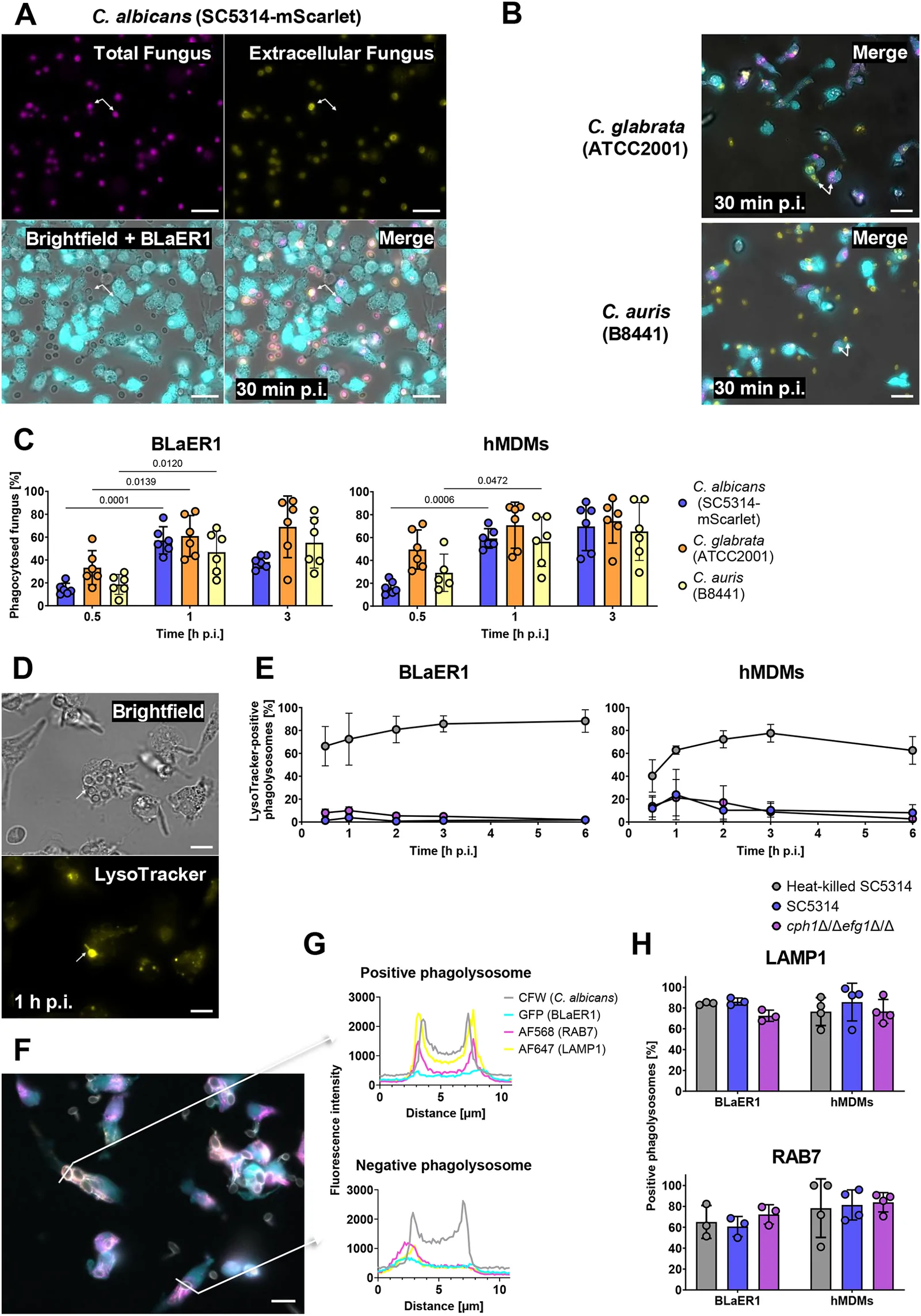
**BLaER1 cells phagocytose *Candida* spp. and form functional phagolysosomes.** (A,B) Phagocytosis of *C. albicans* SC5314-mScarlet WT (A), RBITC-labeled *C. glabrata* ATCC2001 WT (B, top) and *C. auris* B8441 WT (B, bottom) by BLaER1 cells was visualized by live-cell imaging. Extracellular fungal cells were stained with CFW (for *C. albicans* and *C. auris*) or ConA-AF350 (for *C. glabrata*) at 30 min p.i. Double arrows indicate extracellular (mScarlet/RBITC^+^ CFW/ConA-AF350^+^) and intracellular (mScarlet/RBITC^+^ CFW/ConA-AF350^−^) fungal cells. Representative regions are shown, imaged at 400× magnification; image contrast was linearly adjusted to minimum and maximum values. Scale bars: 20 μm. (C) Phagocytosis rates of BLaER1 cells and hMDMs for *C. albicans* SC5314 WT, *C. glabrata* ATCC2001 WT and *C. auris* B8441 WT were quantified by counting all intra- and extracellular fungi in four fields of view across two technical replicates (BLaER1: *n*=6, hMDMs: six donors). (D,E) Acidification of phagolysosomes in BLaER1 cells and hMDMs after infection with *C. albicans* SC5314 WT, heat-killed SC5314 or non-filamentous *cph1*Δ/Δ*efg1*Δ/Δ mutant strains was tracked by live-cell imaging in the presence of LysoTracker for 6 h. (D) Representative image of SC5314 filamentation in an acidified phagolysosome of BLaER1 cells at 1 h p.i. The arrow indicates SC5314 filamentation in an acidic phagolysosome. Image was taken at 400× magnification; image contrast was linearly adjusted to minimum and maximum values. Scale bars: 20 μm. (E) LysoTracker^+^ phagolysosomes were determined as percentage of total phagosomes. Data are displayed as mean±s.d. and were analyzed with a two-way ANOVA and Bonferroni's multiple comparisons test (BLaER1: *n*=3; hMDMs: 4 donors). (F) RAB7^+^ (stained with Alexa Fluor 568; magenta) and LAMP1^+^ (stained with Alexa Fluor 647; yellow) SC5314-containing phagolysosomes 1 h p.i. were visualized by immunofluorescence microscopy. Total fungus was stained with CFW. BLaER1 cells were visualized by GFP expression. Representative regions are shown, imaged at 400× magnification; image contrast was linearly adjusted to minimum and maximum values. Scale bar: 10 μm. (G) Fluorescence intensity profile graphs of each channel across representative marker-positive (top) and marker-negative (bottom) phagolysosomes were plotted from the micrograph in F at the line transects shown. (H) LAMP1^+^ and RAB7^+^ phagolysosomes in BLaER1 cells and hMDMs infected with *C. albicans* SC5314 WT, heat-killed SC5314 or non-filamentous *cph1*Δ/Δ*efg1*Δ/Δ mutant strains were determined microscopically by counting 76 randomly selected phagolysosomes per replicate on average (BLaER1: *n*=3; hMDMs: 4 donors). Data are displayed as mean±s.d. and were analyzed with a two-way ANOVA and Tukey's multiple comparisons test (C,H). Only *P*-values below 0.05 are indicated.

Phagocytosis is the prerequisite for the primary mechanism of pathogen clearance by macrophages: entrapment in a specialized compartment, the phagolysosome (Jia et al., 2024). Phagocytosis of BLaER1 cells and hMDMs was analyzed by differential inside-outside staining (Olivier et al., 2022). Both host cell types were infected with fluorescent fungal cells, either mScarlet-expressing *C. albicans* or rhodamine B-isothiocyanate (RBITC)-labeled *C. glabrata* or *C. auris*. Subsequently, the membrane-impermeable cell wall stains Calcofluor White (CFW; *C. albicans* and *C. auris*) or concanavalin A-Alexa Fluor 350 (ConA-AF350; *C. glabrata*) were added at the corresponding timepoint to stain unphagocytosed fungal cells. We quantified phagocytosis rates microscopically, as the fraction of phagocytosed (fully intracellular) fungal cells (mScarlet/RBITC^+^ CFW/ConA-AF350^−^) among all fungal cells (mScarlet/RBITC^+^ CFW/ConA-AF350^+^). Both cell types were able to rapidly engulf *C. albicans*, *C. glabrata* and *C. auris* (Fig. 3A,B). In BLaER1 cells, phagocytosis rates for *C. glabrata* and *C. auris* were still low 30 min post-infection (p.i.), at about 33% and 18%, respectively (Fig. 3C). After 1 h p.i., phagocytosis rates had increased to over 60% for *C. glabrata* and 46% for *C. auris* in BLaER1 cells (Fig. 3C, Fig. S2B).

After 3 h p.i., the phagocytosis rate remained stable for both species (Fig. 3C). In hMDMs, phagocytosis of *C. glabrata* and *C. auris* followed the same trend, but rates were marginally higher than in BLaER1 cells (Fig. 3C).

For *C. albicans*, phagocytosis rates reached 14% on average 30 min p.i. in BLaER1 cells and increased to 57% 1 h p.i. (Fig. 3C). After 3 h, phagocytosis rates dropped to 38% in BLaER1 cells. In hMDMs, phagocytosis rates were comparable to BLaER1 cells for *C. albicans* after 30 min and 1 h p.i. (Fig. 3C). However, at 3 h p.i. phagocytosis of *C. albicans* by hMDMs had still progressed compared to the 1-h timepoint, in contrast to BLaER1 cells. To better understand these dynamics, we further characterized phagolysosome maturation following *C. albicans* infection of both cell types.

Live-cell imaging in the presence of the acidification marker LysoTracker showed that, following phagocytosis of heat-killed *C. albicans* SC5314 WT, BLaER1 cells progressively acidified their phagolysosomes (Fig. 3D). The number of acidic phagolysosomes increased from 66% at 30 min p.i. to 88% at 6 h p.i. (Fig. 3E). In contrast, uptake of living SC5314 cells resulted in significantly reduced phagolysosome acidification at all timepoints analyzed (Fig. 3E). Filamentation of *C. albicans* was observed in acidified phagolysosomes (Fig. 3D). We therefore tested whether phagosomal acidification is associated with the ability of *C. albicans* to filament by using a non-filamentous mutant strain (*cph1*Δ/Δ*efg1*Δ/Δ) (Wartenberg et al., 2014). Cph1 and Efg1 are key transcription factors that regulate hypha formation (Lo et al., 1997). Phagolysosome acidification was also efficiently reduced by this strain, indicating that the underlying mechanism is filamentation independent and rather relies on the fungal cell wall, as suggested before (Bain et al., 2014). In comparison, hMDMs were also able to generally acidify heat-killed *C. albicans-*containing phagolysosomes, but to a lesser degree than BLaER1 cells (Fig. 3E). Following infection with live SC5314 and *cph1*Δ/Δ*efg1*Δ/Δ strains, phagolysosome acidification was also markedly reduced compared to heat-killed SC5314 in hMDMs (Fig. 3E). However, we found the proportion of LysoTracker^+^ phagolysosomes containing live fungal cells of hMDMs to be consistently higher in comparison to BLaER1 cells. Thus, while BLaER1 cells have a high capacity to acidify phagolysosomes in general, the ability of *C. albicans* to block phagolysosome acidification is more pronounced in these cells than in hMDMs.

In expansion, we analyzed molecular markers of late endosomes, RAB7 and LAMP1, which are characteristic for phagolysosome maturation (Jia et al., 2024; Walpole et al., 2018). Indeed, the majority of *C. albicans-*containing phagolysosomes were positive for both markers at 1 h p.i. in both BLaER1 cells and hMDMs (Fig. 3H).

**Fig. 4.**
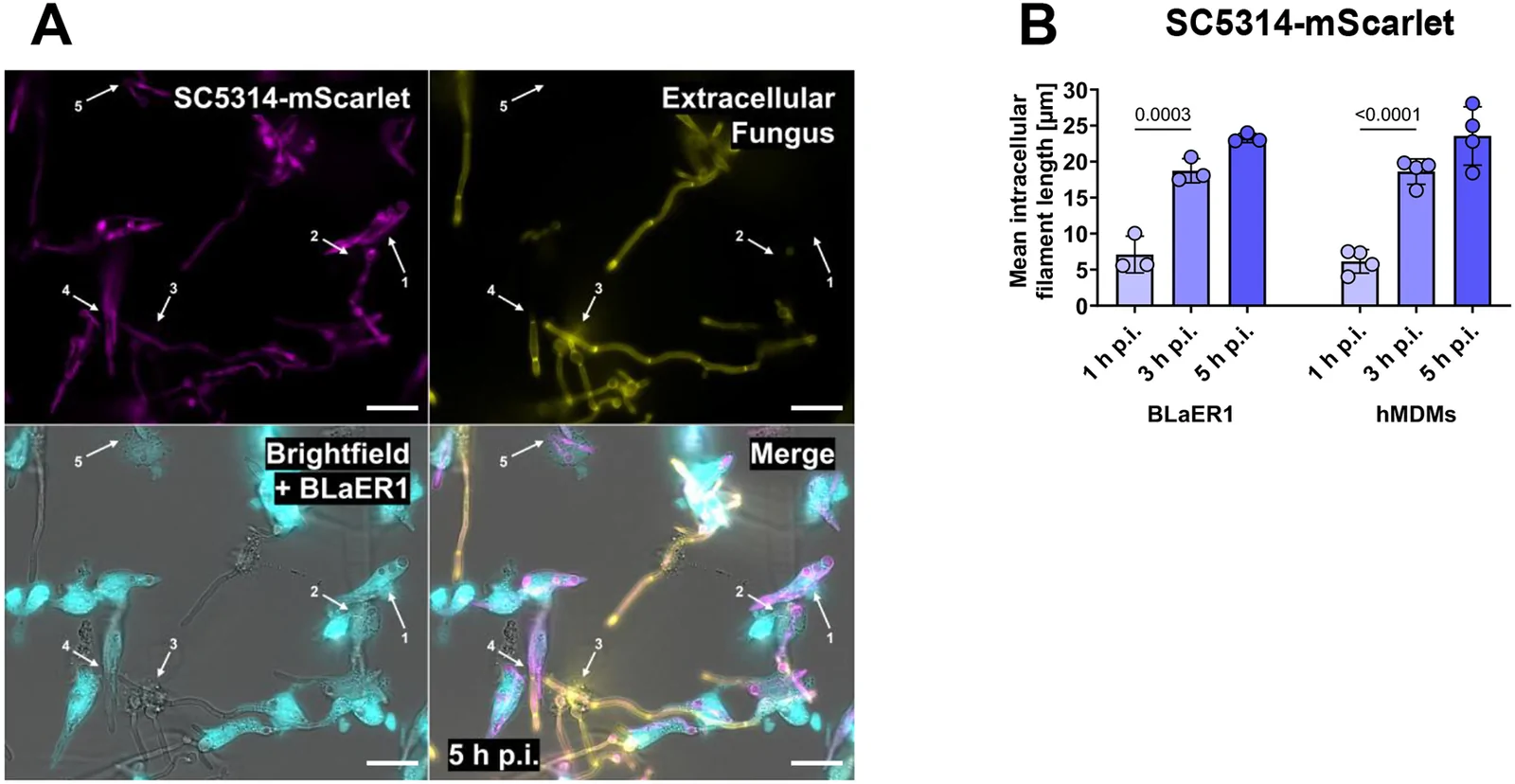
***Candida albicans* can filament intracellularly and escape.** (A) Intracellular filamentation and escape of *C. albicans* SC5314-mScarlet WT was visualized by live-cell imaging after the addition of CFW 5 h p.i. Arrows indicate: fully intracellular hypha (1), killed *C. albicans* cell (2), fully extracellular hypha (3), escaping hypha (4), hyphal folding (5). Images were taken at 400× magnification; image contrast was linearly adjusted to minimum and maximum values. Scale bars: 20 μm. (B) The length of at least 40 fully intracellular hyphae was measured microscopically 1, 3 and 5 h p.i. (*n*=3). Data are displayed as mean±s.d. and were analyzed with a one-way ANOVA and Tukey's multiple comparisons test. Only *P*-values below 0.05 are indicated.

Early initial hypha formation of *C. albicans* in BLaER1 cells was visible already after 1 h (Fig. S2A) and hyphal extension occurred within 3 h p.i. (Fig. S2A). Filamentous growth of *C. albicans* can permeabilize the phagolysosome and host cell membrane (Wartenberg et al., 2014; Westman et al., 2018). As a consequence, the extracellular dye CFW might enter the host cell, explaining the apparently decreased phagocytosis rate. We therefore analyzed intracellular filamentation and *C. albicans* escape in more detail at 5 h p.i. Indeed, extended filaments were visible in BLaER1 cells and different stages of host cell exit could be observed (Fig. 4A): fully intracellular hyphae (arrow 1), dead (mScarlet^−^) and fully intracellular (CFW^−^) yeast cells (arrow 2), hyphae that invade and kill another BLaER1 cell (arrow 3), escaping hyphae (arrow 4) and hyphal folding (arrow 5) could be detected. Fully intracellular filament lengths were highly similar between BLaER1 cells and hMDMs at all timepoints (Fig. 4B).

Intracellular hyphae extended significantly between 1 and 3 h p.i., but growth slowed down after 3 h p.i. (Fig. 4B). Around BLaER1 cells, overall hyphae did visibly grow more (Fig. 4A), supporting the notion that *C. albicans* escape begins around 3 h p.i. In contrast, phagocytosis of *C. albicans* progressed in hMDMs between 1 and 3 h p.i. (Fig. 3C). This suggests that hMDMs contain hyphal growth better than BLaER1 cells, which might be partially explained by the more acidic milieu of *C. albicans*-containing phagolysosomes in hMDMs.

In conclusion, BLaER1 cells are highly competent at phagocytosis, and phagolysosome maturation. However, they show a reduced ability to control hypha formation of surviving fungal cells compared to hMDMs.

### BLaER1 cells and hMDMs exhibit a similar, early pro-inflammatory immune response to *C. albicans* infection

Intracellular filamentation of *C. albicans* is a major escape mechanism that ultimately results in killing of the host cell (Lo et al., 1997; Wartenberg et al., 2014). The induction of macrophage cell death by *C. albicans* is known to be a two-step process *in vitro* (Uwamahoro et al., 2014). The early phase (up to ≈6 h p.i.) is initiated by phagosomal escape (Westman et al., 2018), candidalysin secretion (Kasper et al., 2018) and pro-inflammatory cell death, mostly pyroptosis (Uwamahoro et al., 2014; Wellington et al., 2014), which can be induced by both filamentation-dependent and -independent factors (O'Meara et al., 2018). This is followed by a stationary phase, and finally a late stage. This late stage is driven by competition for glucose between the macrophage and *C. albicans* (Tucey et al., 2018), other forms of programmed cell death, such as necroptosis and apoptosis (Banoth et al., 2020; Li et al., 2021), the general cell death factor NINJ1 (Weerasinghe et al., 2026), and, finally, the physical forces of growing hyphae (Uwamahoro et al., 2014).

**Fig. 5.**
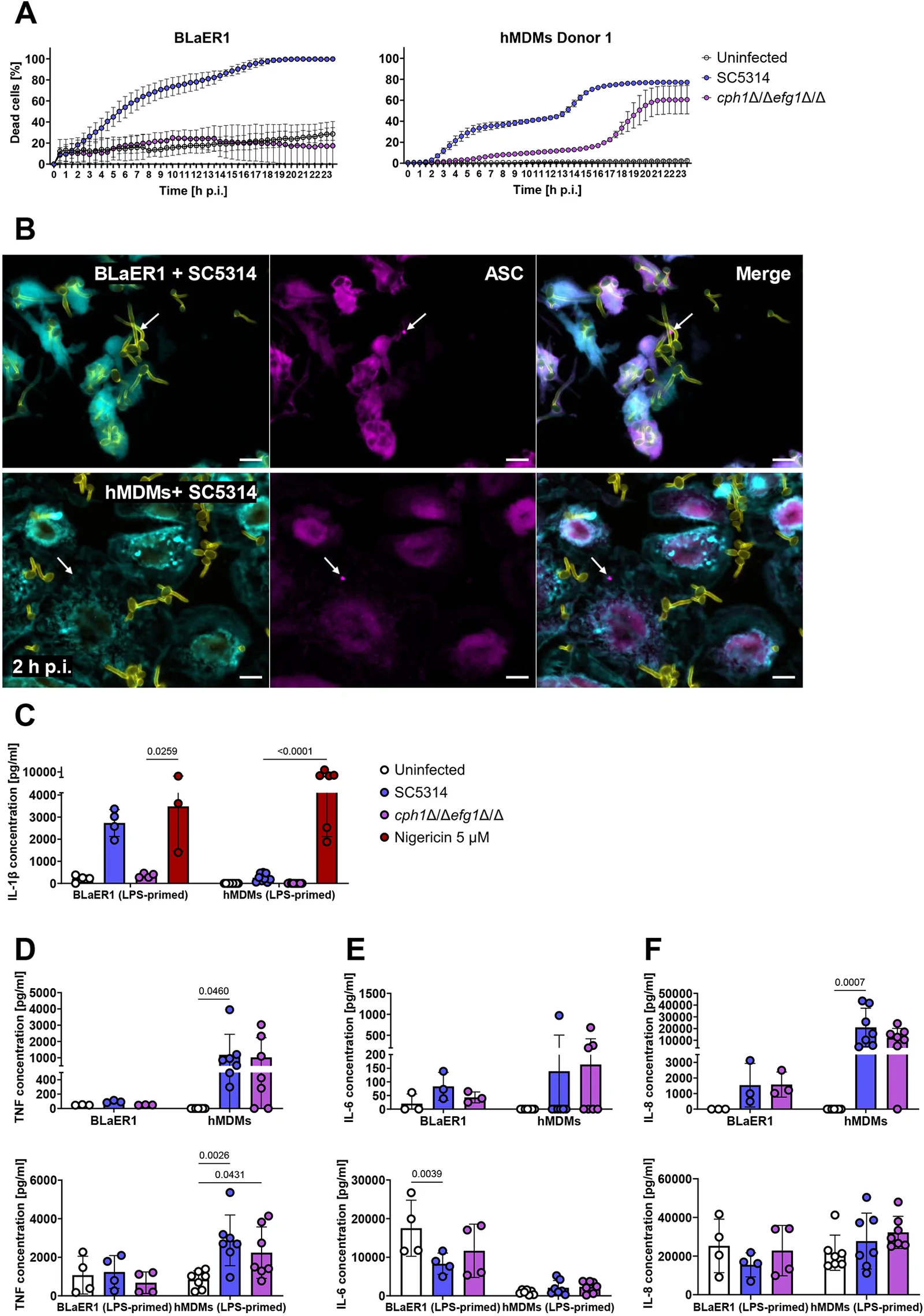
**BLaER1 cells exhibit early pro-inflammatory immune responses that are comparable to hMDMs.** (A) Left: Cell death of uninfected BLaER1 cells and those infected with *C. albicans* SC5314 WT or with the non-filamentous *cph1*Δ/Δ*efg1*Δ/Δ mutant strain was tracked by live-cell imaging as loss of GFP signal. Cell numbers were normalized to the first timepoint (*n*=5). Right: Cell death of hMDMs either uninfected or infected with the same strains was tracked by live-cell imaging in presence of the membrane-impermeable dye SYTOX Green. Total cell number was determined based on SYTO Deep Red^+^ nuclei and normalized to the first timepoint (one donor; error bars represent three technical replicates). (B) ASC speck formation (stained with Alexa Fluor 647, magenta) in LPS-primed BLaER1 cells as well as LPS-primed hMDMs was visualized by immunofluorescence microscopy 2 h p.i. with *C. albicans* SC5314 WT. Total fungus was stained with CFW (yellow). Cytoskeleton of hMDMs was stained with phalloidin-Alexa Fluor 488 (cyan). Representative regions are shown, taken at 400× magnification; contrast was linearly adjusted to minimum and maximum values. Scale bars: 10 μm. (C) Secretion of IL-1β from LPS-primed BLaER1 cells and LPS-primed hMDMs was quantified by ELISA 5 h p.i. with the same strains as in A or treated with 5 μM nigericin as positive control (BLaER1: *n*=4; hMDMs: 9 donors). (D-F) Secretion of the pro-inflammatory cytokines TNF (D), IL-6 (E) and IL-8 (F) from unprimed and LPS-primed BLaER1 cells and hMDMs was quantified by ELISA 24 h after infection with the same strains as in A (BLaER1: *n*=3 and 4; hMDMs: 7 donors). Values of 0 pg/ml represent ‘not detected’. Data are displayed as mean±s.d. and were analyzed with a two-way ANOVA and Tukey's multiple comparisons test. Only *P*-values below 0.05 are indicated.

We quantified the percentage of dead phagocytes by live-cell imaging for 24 h after infection with SC5314 WT and *cph1*Δ/Δ*efg1*Δ/Δ. In BLaER1 cells, the initial phase of cell death was very pronounced, increasing rapidly to over 60% dead host cells within 8 h of SC5314 infection (Fig. 5A). BLaER1 cell death slowed down between 8 and 13 h p.i., but a steady increase could still be detected. Eventually, after 19 h, all BLaER1 cells were killed by the WT strain. In hMDMs, the initial phase of cell death lasted only until 6 h after SC5314 infection (Fig. 5A) and was less pronounced, with ∼35% of hMDMs being killed. However, a strong donor variability was observed (Fig. 5A, Fig. S3A). In contrast to the BLaER1 cells, following a 13-h period of infection, a second phase of cell death commenced in hMDMs (Fig. 5A). This process plateaued after 16-18 h of infection, resulting in ∼75% host cell killing. The kinetics were again strongly donor dependent (Fig. S3A). ASC speck formation, an established readout for NLRP3 inflammasome activation (Stutz et al., 2013), was visible in both BLaER1 cells and hMDMs after 2 h of *C. albicans* infection (Fig. 5B). This matches the central dogma in the field that early *C. albicans*-induced cell death in macrophages is mainly pyroptosis driven (Uwamahoro et al., 2014; Wellington et al., 2014).

Infection with the non-filamentous *cph1*Δ/Δ*efg1*Δ/Δ strain did not result in substantial early cell death compared to the uninfected control in either host cell (Fig. 5A). However, starting from 16 h p.i., a significant increase in host cell death was observed in hMDMs infected with *cph1*Δ/Δ*efg1*Δ/Δ (Fig. 5A, Fig. S3A). Collectively, BLaER1 cells are, therefore, most effective at modeling the initial phase of macrophage infection. Furthermore, the process of their cell death appears to be wholly contingent upon fungal filamentation, as opposed to hMDMs.

We quantified IL-1β secretion following LPS priming to assess the early activation of the inflammasome, a process connected to pyroptotic cell death (Bergsbaken et al., 2009). In agreement with the cell death kinetics, significant IL-1β secretion was detected by both BLaER1 and hMDMs upon SC5314 infection after 5 h (Fig. 5C). While the amount of SC5314-induced IL-1β release reached almost the level of nigericin treatment (positive control) for BLaER1 cells (Fig. 5C), it remained significantly lower for hMDMs (Fig. 5C), mirroring their respective degrees of early phase cell death (Fig. 5A). In contrast, no significantly increased amount of IL-1β was secreted by both host cells upon infection with *cph1*Δ/Δ*efg1*Δ/Δ compared to uninfected controls (Fig. 5C). Interestingly, no IL-1β release was detected from uninfected hMDMs, while uninfected BLaER1 cells secreted low levels, reflecting the baseline cell death observed in each model (Fig. 5A).

Lactate dehydrogenase (LDH) release after 24 h p.i., a measure of host cell damage (Kasper et al., 2018), followed the same pattern as IL-1β secretion, and remained unaffected by priming (Fig. S3B). The concentration of LDH secreted by BLaER1 cells was about twice as high as for hMDMs, directly reflecting the difference in host cell number per well.

Over the course of infection, macrophages also secrete pro-inflammatory cytokines, such as TNF, IL-6 and IL-8, which further drive and orchestrate the immune response against pathogens (Wynn et al., 2013). After 24 h of *C. albicans* infection, the secretion of TNF from unprimed BLaER1 cells was found to be lower by approximately one order of magnitude in comparison to unprimed hMDMs, despite the higher number of BLaER1 cells per condition (Fig. 5D, top). Only the filamentous SC5314 strain was able to induce an increased TNF release compared to the uninfected controls in unprimed BLaER1 cells (Fig. 5D). In contrast, *C. albicans* infection in unprimed hMDMs triggered TNF release, independently of the ability of the strain to filament (Fig. 5D). For IL-6, both unprimed BLaER1 cells and hMDMs released similarly low levels upon infection (Fig. 5E, top). For the few hMDM donors for which IL-6 was measurable, it was detected for both SC5314 and *cph1*Δ/Δ*efg1*Δ/Δ (Fig. 5E). IL-8 secretion from unprimed and infected BLaER1 cells and hMDMs was detected regardless of the ability of the *C. albicans* strain to filament (Fig. 5F, top). Similar to TNF, the concentration of IL-8 released by the unprimed BLaER1 cells was approximately one order of magnitude lower than that secreted by unprimed hMDMs. In summary, non-primed BLaER1 cells are able to secrete cytokines in response to a strong trigger such as filamenting *C. albicans*, but do so significantly less than hMDMs.

Overall, LPS priming increased cytokine secretion in all cases for both cell types (Fig. 5D-F, bottom panels). In BLaER1 cells, the level of secreted cytokines reached a level comparable to that of  hMDMs, showing that the cells respond very well to bacterial pathogen-associated molecular patterns, independently of *C. albicans* infection.

In the host, pathogens that reach the bloodstream can be opsonized by antibodies or complement factors, enhancing the recognition by innate immune cells (Wellington et al., 2003, 2007). In line with this, opsonization of *C. albicans* with human serum led to increased IL-1β (Fig. S3C) and TNF (Fig. S3D) secretion from both host cell types, but did not affect host cell damage (Fig. S3E).

Overall, these data demonstrate that BLaER1 cells replicate key features of hMDMs, including efficient phagocytosis, intracellular killing of different *Candida* species, and functional phagolysosome maturation. They best resemble hMDMs during the early phase of *C. albicans* infection, recapitulating aspects of pro-inflammatory cell death driven by filamentation and filamentation-associated factors. This establishes BLaER1 cells as a physiologically relevant model for mechanistic studies.

### Candidalysin and gasdermin D are the key factors leading to IL-1β release and host cell death

Candidalysin is a cytolytic peptide toxin encoded by the gene *ECE1* (extent of cell elongation 1) that is secreted by hyphae (Moyes et al., 2016). In phagocytes primed with LPS or β-glucan, it leads to NLRP3 inflammasome activation via K^+^-efflux from the cytosol (Kasper et al., 2018). This is likely a consequence of toxin-mediated plasma membrane destabilization resulting in release of IL-1β and host cell death independently of pyroptosis (Kasper et al., 2018). Following NLRP3 inflammasome activation, gasdermin D (GSDMD) undergoes proteolytic cleavage (Devant and Kagan, 2023). The N-terminal fragment forms a pore in the host cell membrane, leading to the release of IL-1β and pyroptosis (Devant and Kagan, 2023). Therefore, to determine the usefulness of knockout BLaER1 cells to study interactions with fungi, we employed *GSDMD^−/−^* BLaER1 cells (Volkmar et al., 2024) to analyze the role of this host factor in *C. albicans*-induced cell death in humans. Mouse cells lacking components of the NLRP3 inflammasome and GSDMD have been used before to decipher the role of these factors as well as candidalysin in *C. albicans*-induced host cell death (Ding et al., 2021; Kasper et al., 2018; Olivier et al., 2022). However, the direct effects of gasdermin D and candidalysin have not been elucidated so far for human cells.

**Fig. 6.**
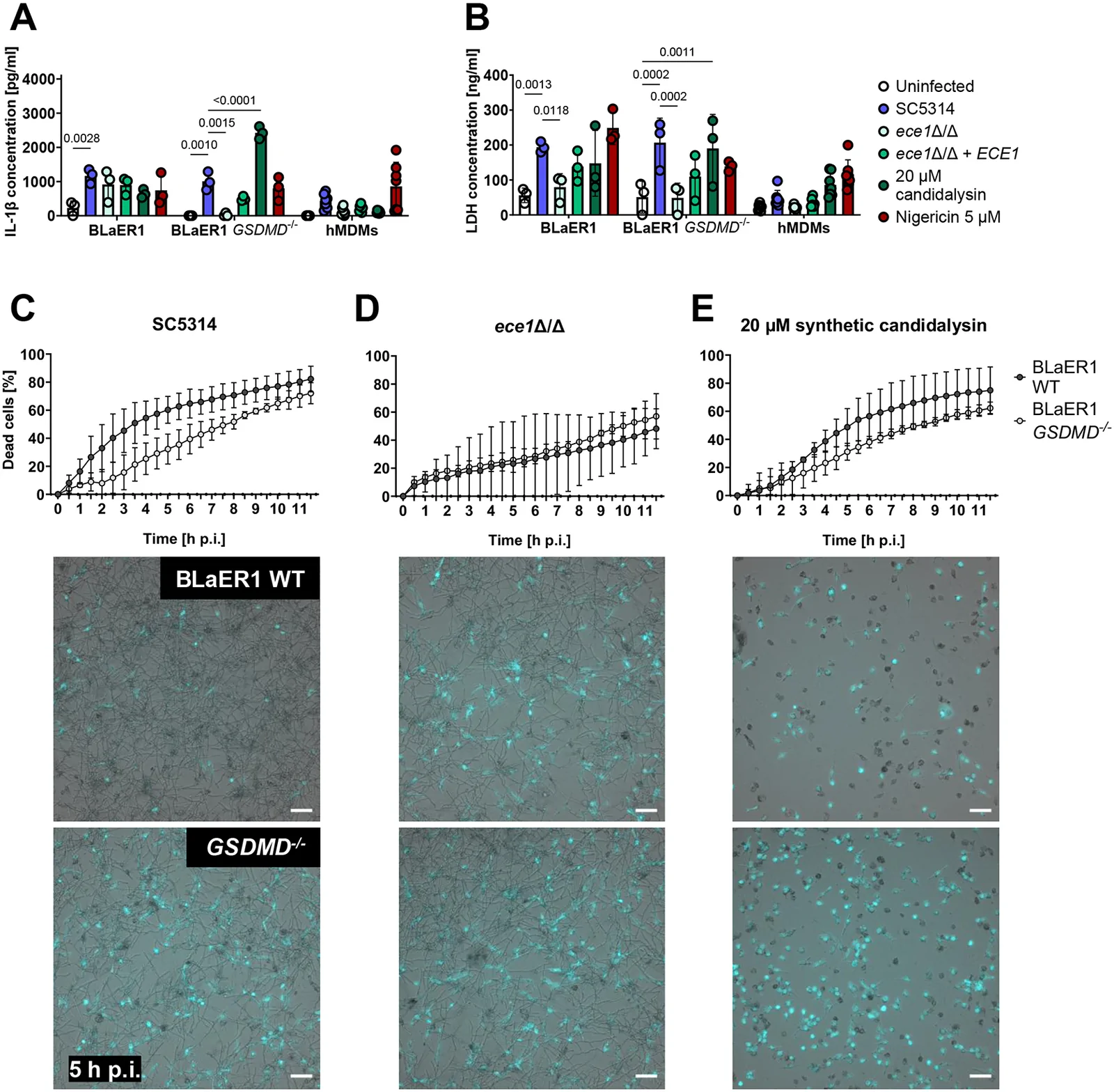
**Candidalysin and gasdermin D induce IL-1β release and host cell death.** (A) Secretion of IL-1β from LPS-primed BLaER1 WT and *GSDMD^−/−^* cells (left) as well as LPS-primed hMDMs (right) was quantified by ELISA 5 h after infection with *C. albicans* SC5314 WT, *ece1*Δ/Δ or *ece1*Δ/Δ*+ECE1* mutant strains or treatment with 20 μM synthetic candidalysin or 5 μM nigericin as positive control (BLaER1: *n*=3; hMDMs: 7 donors). (B) Release of LDH from LPS-primed BLaER1 WT and *GSDMD^−/−^* cells (left) as well as LPS-primed hMDMs (right) was quantified 5 h after infection and treatment as in A (BLaER1: *n*=3; hMDMs: 7 donors). (C-E) Cell death of LPS-primed BLaER1 WT and *GSDMD^−/−^* cells, infected with *C. albicans* WT (C) or the *ece1*Δ/Δ mutant strain (D) or after treatment with 20 μM synthetic candidalysin (E) was tracked for 12 h by live-cell imaging as loss of GFP signal. Cell numbers were normalized to the first timepoint. Images below each graph show representative regions from the 5-h timepoint of the corresponding infection of treatment. Images taken at 100× magnification; contrast was linearly adjusted to minimum and maximum values. Scale bars: 50 μm (*n*=3). Data are displayed as mean±s.d. and were analyzed with a one-way ANOVA and Tukey's multiple comparisons test (A,B, hMDMs) or two-way ANOVA and Tukey's multiple comparisons test (A,B, BLaER1 cells) or Šídák's multiple comparisons test (C-E). Only *P*-values below 0.05 are indicated.

Infection of LPS-primed BLaER1 WT cells and hMDMs with *ece1*Δ/Δ, a mutant strain that lacks the pre-pro-protein of candidalysin, Ece1 (Moyes et al., 2016), showed a slightly reduced IL-1β release 5 h p.i. in both host cell types compared to infection with SC5314 (Fig. 6A). Treatment with synthetic candidalysin also led to an increased IL-1β release compared to the uninfected control (Fig. 6A). In *GSDMD^−/−^* BLaER1 cells after SC5314 infection, IL-1β was slightly but not significantly lower compared to WT BLaER1 cells (Fig. 6A). Remarkably, virtually no IL-1β release was detected upon infection of *GSDMD^−/−^* BLaER1 cells with *ece1*Δ/Δ. This was partially reversible upon infection with a strain in which one copy of *ECE1* has been re-introduced (*ece1*Δ/Δ*+ECE1*) in *GSDMD^−/−^* BLaER1 cells. NLRP3 inflammasome activation was visible in *GSDMD^−/−^* BLaER1 cells in the form of ASC speck formation (Stutz et al., 2013) (Fig. S4A). Synthetic candidalysin treatment led to a substantial release of IL-1β across all replicates (Fig. 6A). This suggests that gasdermin D and candidalysin, but not host cell lysis via filamentation per se, are responsible for the early IL-1β release. In accordance with previously published results (Kasper et al., 2018), TNF secretion was independent of synthetic candidalysin treatment (Fig. S4B).

We previously engineered a non-filamentous *cph1*Δ/Δ*efg1*Δ/Δ strain that constitutively expresses and secretes candidalysin in the yeast form (*cph1*Δ/Δ*efg1*Δ/Δ-CaL-OE) (Mogavero et al., 2021), which was unable to rapidly escape from the phagolysosome (Westman et al., 2018). Infection with this strain did not induce increased IL-1β release compared to the original *cph1*Δ/Δ*efg1*Δ/Δ mutant strain in any host cell type (Fig. S4C). To exclude additional Cph1/Efg1-associated effects, we tested another strain with defective hypha formation, *eed1*Δ/Δ, which can initiate filamentation, but is unable to maintain filament elongation and reverts to the yeast form (Martin et al., 2011). This results in native candidalysin secretion, albeit at a lesser extent than the WT (Mogavero et al., 2021).

As expected, *eed1*Δ/Δ formed short hyphae in the phagolysosome of BLaER1 cells and hMDMs at 5 h p.i. (Fig. S4D), unlike *cph1*Δ/Δ*efg1*Δ/Δ-CaL-OE (Fig. S4D). In BLaER1 WT and *GSDMD^−/−^* cells, *eed1*Δ/Δ did not result in increased IL-1β secretion compared to *cph1*Δ/Δ*efg1*Δ/Δ-CaL-OE infection (Fig. S4B). IL-1β release of *eed1*Δ/Δ-infected BLaER1 *GSDMD^−/−^* cells was reduced compared to BLaER1 WT cells, suggesting that the detected IL-1β secretion was mostly GSDMD dependent. In hMDMs, in which no baseline IL-1β was detected, *eed1*Δ/Δ resulted in slightly, but not statistically significantly, increased IL-1β release compared to *cph1*Δ/Δ*efg1*Δ/Δ infection. This intermediate phenotype could be due to a transient rupture of phagolysosomes during initial hypha formation (Westman et al., 2018). Thus, although fungal filamentation is not required to induce IL-1β release from the host cells (O'Meara et al., 2018), hyphae seem to be necessary to deliver candidalysin to the host cell cytosol. Interestingly, no IL-1β release was measurable in uninfected *GSDMD^−/−^* BLaER1 cells (Fig. 6A), indicating that gasdermin D is required for LPS-induced IL-1β secretion.

LDH release was similar between BLaER1 WT and *GSDMD^−/−^* cells following infection with SC5314 at 5 h (Fig. 6B). In hMDMs, the level of LDH release was significantly lower (Fig. 6B), reflecting the reduced rates of cell death at this timepoint compared to WT BLaER1 cells (Fig. 5A, Fig. S3A). Interestingly, SC5314-induced cell death was significantly lower in *GSDMD^−/−^* cells than in WT cells between 2 to 6 h p.i. (Fig. 6C), despite a comparable LDH release (Fig. 6B). Upon infection with the *ece1*Δ/Δ strain, a lower LDH release was visible in hMDMs, as well as BLaER1 WT and *GSDMD^−/−^* cells, although the effect was less pronounced in the BLaER1 WT cells (Fig. 6B). Furthermore, early cell death was decreased after *ece1*Δ/Δ infection for both WT and *GSDMD^−/−^* BLaER1 cells (Fig. 6D) compared to SC5314 infection (Fig. 6C). There was no difference between the host cell lines (Fig. 6D), again reflecting the LDH release (Fig. 6B). Both fungal strains showed significant filamentation in BLaER1 and *GSDMD^−/−^* cells (Fig. 6C,D). The physical forces and metabolic activity of *C. albicans* hyphae are likely responsible for the observed candidalysin- and gasdermin D-independent host cell death, as described previously (Tucey et al., 2018; Uwamahoro et al., 2014). Alternative forms of programmed cell death might also be responsible for the residual cell death (Banoth et al., 2020; Li et al., 2021), since the necessary components are stably expressed by LPS-primed BLaER1 cells (and hMDMs; Fig. 2A). Finally, synthetic candidalysin also induced cell death to a similar degree in WT and *GSDMD^−/−^* BLaER1 cells (Fig. 6E), reaching levels that were comparable with SC5314 infection (Fig. 6C). This matches the LDH data (Fig. 6B). In conclusion, LDH release at 5 h p.i. generally correlated with *C. albicans*-induced cell death but not directly with the presence of gasdermin D. Importantly, exposure to candidalysin could lead to LDH release independently of gasdermin D.

Taken together, these data show an uncoupling of IL-1β secretion and host cell death. The former seems to be strictly dependent on candidalysin and gasdermin D in BLaER1 cells (Fig. 6A), whereas cell death in this model involves candidalysin (Fig. 6C-E) as well as filamentation and filamentation-associated factors (Fig. 5A).

## DISCUSSION

Macrophage models of *Candida* infection, particularly of human origin, are instrumental in understanding fungal pathogenicity. Good models can enable the identification and dissection of fungal and host factors involved in their interactions. Here, we establish transdifferentiated BLaER1 cells as an effective model of *Candida* infection, and show that they resemble the ‘gold standard’ hMDMs in terms of macrophage-defining traits. This includes morphology, PRR expression, phagocytosis and phagolysosome maturation, inflammasome activation, and early pro-inflammatory cytokine secretion as well as transcriptional reaction to infection. BLaER1 cells respond well to immunomodulatory stimuli, such as LPS priming and opsonization. Finally, using *GSDMD^−/−^* BLaER1 cells, we show for the first time in a human macrophage model, that gasdermin D and candidalysin have distinct roles in triggering pyroptosis and inducing lytic host cell death. These results confirm the dual role of candidalysin that was proposed previously (Kasper et al., 2018).

Like any model system, BLaER1 cells have advantages and limitations that need to be considered for experimental and study design. The constitutive expression of GFP offers a useful viability marker and allows tracking of cell death via flow cytometry or live-cell imaging without the addition of any additional fluorescent dye. This method might even be more robust than using membrane-impermeable markers such as SYTOX Green, since it not only depends on membrane integrity, but also translational activity of the cell. Still, for some assays, the use of *eGFP^−/−^* BLaER1 cells (Volkmar et al., 2024) might give users more freedom of choice for fluorophores. An important limitation of BLaER1 cells is that they are less adherent than hMDMs, potentially resulting in detachment of cells. This can cause unequal cell numbers and empty areas within a well, increasing the variability of the results, may facilitate microbial growth, and may lead to *C. albicans* filamentation. Attempts to avoid this by a reduced number of washing steps could come with the additional challenge of more residual fetal bovine serum (FBS) or LPS in the wells. Importantly, although unprimed BLaER1 cells secreted pro-inflammatory cytokines in response to *C. albicans* infection, cytokine levels were significantly lower than for unprimed hMDMs. This needs to be considered for the potential use in more complex infection models, where cytokines are required to stimulate other cell types. Finally, some suspension cells can remain after transdifferentiation, and although these are mostly washed away prior to infection, it cannot be excluded that residual cells are present, and the proliferative potential of these cells could influence results. Nevertheless, transdifferentiation of BLaER1 cells is highly efficient and the vast majority of B cells routinely convert to macrophage-like cells.

After transdifferentiation, BLaER1 cells were CD14^+^. This is a key similarity to hMDMs, which are positively selected for CD14 prior to differentiation. Furthermore, it also represents an advantage over THP-1 cells. Even with optimized differentiation protocols, THP-1 cells display significantly lower CD14 expression than hMDMs (Liu et al., 2019; Park et al., 2007). Additionally, phorbol-12-myristate 13-acetate, used to induce differentiation of THP-1 cells into macrophages, leads to significant transcriptomic deviations from hMDMs (Liu et al., 2023), unlike BLaER1 cells, which become transcriptionally more similar to hMDMs during transdifferentiation (Rapino et al., 2013).

The expression of macrophage surface markers in comparison between BLaER1 to M-CSF-differentiated hMDMs was assessed in a previous publication by Volkmar et al. (2024). In contrast to our study, the authors detected moderate expression of CD163 in BLaER1 cells and M-CSF-differentiated hMDMs. This discrepancy is likely attributable to differences in the reagents used, but also subtle variations in the isolation and differentiation procedures as both cell types displayed this phenotype within the same laboratory. Furthermore, Volkmar et al. (2024) proposed that BLaER1 cells better resemble GM-CSF-differentiated hMDMs, which display M1 activation features (Reales-Calderón et al., 2014). Similarly, BLaER1 cells in this study also had more M1 characteristics (CD80^+^, TLR2/4^+^, CD163^−^) than M-CSF-differentiated hMDMs. Additionally, LPS priming is described to have a comparable effect to GM-CSF (Reales-Calderón et al., 2014). Transcriptional profiling of LPS-primed BLaER1 cells and hMDMs showed that, despite the remaining transcriptional differences, specific gene sets connected to the execution of programmed host cell death pathways were transcribed at similar levels. Furthermore, the transcriptional response to *C. albicans* infection of both LPS-primed cell types was found to be concordant.

During infection, macrophages detect pathogens via different PRRs (Shapouri-Moghaddam et al., 2018). Both BLaER1 cells and hMDMs expressed a variety of PRRs and rapidly phagocytosed different fungal pathogens. Phagocytosis rates were generally found to be similar to that of THP-1 cells, another human-derived macrophage-like cell line (Liu et al., 2019; Vaz et al., 2019).

Subsequently, BLaER1 cells were able to form mature phagolysosomes. Data for the maturation markers RAB7 and LAMP1 were comparable to other cell lines (Bain et al., 2014; Kasper et al., 2018; Westman et al., 2020).

Like other murine and human macrophages (Case et al., 2023; Ghosh et al., 2009; Silao et al., 2019), BLaER1 cells triggered filamentation of *C. albicans*. Phagocytes contain filamenting *C. albicans* cells to some degree, but biological membranes have a limited capacity to stretch (Westman et al., 2020). Therefore, macrophages fuse cellular lysosomes and initiate lysosome biogenesis (Westman et al., 2020). Eventually, however, growing hyphae can escape *in vitro* (Westman et al., 2020). Intracellular hyphal lengths in BLaER1 cells were similar to those in hMDMs. Nevertheless, escape from BLaER1 cells started earlier than from hMDMs. This suggests that BLaER1 cells have a reduced capacity to control *C. albicans* hyphal growth, and that hyphae beyond a certain size can exit the host cell.

In BLaER1 cells, phagolysosomes containing live *C. albicans* were less frequently acidified than in hMDMs. The acidic pH might inhibit *C. albicans* filamentous growth (Bain et al., 2014), and at least partially explain the striking *C. albicans* filamentation in BLaER1 cells.

Escape of *C. albicans* from macrophages is closely connected to host cell death. The early phase is mostly characterized by inflammasome-mediated IL-1β secretion and pyroptosis (Uwamahoro et al., 2014; Wellington et al., 2014). This was seen very prominently in SC5314-infected BLaER1 cells and can be partially explained by the strong early filamentation as described above. Additionally, filamentation-independent triggers of host cell death are known (O'Meara et al., 2018). Accordingly, late stage, *cph1*Δ/Δ*efg1*Δ/Δ-induced cell death of hMDMs was observed (this study), but the same was not detected in BLaER1 cells. In contrast, uninfected BLaER1 cells displayed low levels of cell death. Photobleaching of GFP, which cannot be replenished from the surrounding medium like SYTOX Green, might at least partially be detected as cell death, and explain this baseline in uninfected BLaER1 cells.

Unprimed BLaER1 cells only secreted elevated levels of the pro-inflammatory cytokines TNF and IL-6 upon infection with the SC5314 WT but not the non-filamentous *cph1*Δ/Δ*efg1*Δ/Δ. In hMDMs, however, secretion of cytokines was not only significantly higher, but also depended less on hypha formation. LPS priming increased the levels of cytokine secretion in all tested conditions. The responsiveness of BLaER1 cells to LPS confirms the functionality of TLR4-dependent signaling. This is a clear advantage over other human cell lines such as THP-1, in which this signaling is defective (Bosshart and Heinzelmann, 2016). Opsonization of pathogens with serum components such as complement or IgG represents an additional mechanism to facilitate innate immune responses (Wellington et al., 2003, 2007). Accordingly, both BLaER1 cells and hMDMs increased cytokine secretion significantly upon opsonization of *C. albicans*.

The hypha-associated cytolytic peptide toxin candidalysin is a key virulence factor of *C. albicans*. It is responsible for causing damage of epithelial cells during mucosal infections (Moyes et al., 2016), but is also required for commensal growth (Fróis-Martins et al., 2025; Liang et al., 2024). In macrophages, candidalysin can activate the NLRP3-inflammasome by causing K^+^ efflux from the cytosol (Kasper et al., 2018), likely as a consequence of its cytolytic properties.

However, candidalysin plays only a minor role in *C. albicans*-induced pyroptosis and rather causes pyroptosis-independent cytolytic cell death in human phagocytes (Kasper et al., 2018). Causing host cell death to escape from the phagocyte likely provided an evolutionary benefit for *C. albicans*, but activation of pyroptosis also enables the host to mount a more efficient immune response (Drummond et al., 2019; König et al., 2020). In studies with mouse models of *C. albicans* infection, it was found that candidalysin and gasdermin D, the terminal factor for the execution of pyroptosis, contribute to host cell death and escape of *C. albicans* (Ding et al., 2021; Olivier et al., 2022). In mice, IL-1β release is known to be markedly reduced in *Gsdmd^−/−^* macrophages after *C. albicans* infection and IL-1β secretion is still detectable upon infection of *Gsdmd^−/−^* macrophages with an *ece1*Δ/Δ mutant strain (Ding et al., 2021). Necrosulfonamide (NSA), an inhibitor of gasdermin D oligomerization, has been shown to result in a significant reduction of IL-1β release in hMDMs (Ding et al., 2021). NSA, however, additionally suppresses necroptosis by inhibiting MLKL (Dong et al., 2017). This complicates any conclusions about the direct role of gasdermin D.

In contrast, we found no difference in IL-1β secretion between WT and *GSDMD^−/−^* BLaER1 cells. We also detected almost no IL-1β release upon infection of *GSDMD^−/−^* cells with *ece1*Δ/Δ. In human macrophages, gasdermin D and candidalysin appear as the two determining factors for *C. albicans*-induced, early IL-1β secretion. Deletion of either component alone was compensated for by the other. Thus, the use of *GSDMD^−/−^* BLaER1 cells allowed us to dissect the redundant functions of candidalysin and gasdermin D for the first time in a human macrophage model.

Synthetic candidalysin induced *C. albicans* WT-like IL-1β secretion, LDH release and host cell death in both WT and *GSDMD^−/−^* BLaER1 cells. We therefore propose that the toxin can in principle facilitate all three events independently of gasdermin D. The mechanisms are potentially similar to candidalysin-induced activation of the NLRP3-inflammasome through K^+^ efflux: the toxin destabilizes the plasma membrane, leading to the release of these host proteins (Kasper et al., 2018). The mode of action of candidalysin is likely concentration dependent (Russell et al., 2022).

While *ECE1* expression and candidalysin secretion correlate with hyphal length (Birse et al., 1993; Moyes et al., 2016), the exact concentration of candidalysin secreted by *C. albicans* in a given native environment is unknown. Secretion into compartments such as the phagolysosome (Kasper et al., 2018) may lead to increased local concentrations. Previously, it was shown that candidalysin expression without filamentation is not enough to allow *C. albicans* to escape from the phagolysosome (Westman et al., 2018), indicating that this membrane cannot be damaged by the toxin alone. Our model supports this, as candidalysin expression without egress from the phagolysosome was not sufficient to induce IL-1β secretion. Thus, similar to invasion of epithelial cells by *C. albicans* (Mogavero et al., 2021), delivery of the toxin into the cytosol is required. The intermediate phenotypes observed for the *eed1*Δ/Δ mutant regarding intraphagosomal hypha formation as well as induced IL-1β release in hMDMs further support this hypothesis.

In light of the threat of fungal infections through emerging pathogens such as *C. auris* and the increase of antifungal resistance, new tools to study host–fungus interactions are desperately needed. We have shown here that BLaER1 cells can be employed and genetically engineered to identify and characterize host factors of *Candida–*macrophage interactions. This new cellular tool could improve our understanding of fungal disease and may open new avenues for the development of antifungal treatment strategies.

## MATERIALS AND METHODS

### Ethics statement

Blood was obtained from healthy human volunteers with written informed consent. The procedures for blood donation and the subsequent use for this study were approved by the Jena institutional ethics committee (Ethik-Kommission des Universitätsklinikums Jena, Permission No 2207-01/08).

### BLaER1 culture and transdifferentiation

The BLaER1 cell line (Table S2) was previously generated as described by Rapino et al. (2013) and cultured and transdifferentiated according to the published protocol, with minor modifications to cell numbers. BLaER1 *GSDMD^−/−^* cells (Table S2) were generated by Volkmar et al. (2024) following the method described by Vierbuchen et al. (2017).

BLaER1 cells were maintained in BLaER1 medium (Tables S4, S5) at 37°C, 5% CO_2_. Cells were passaged every 2-3 days at a split ratio of 1:3 to 1:5 and were used for experiments up to a maximum of 30 passages.

For transdifferentiation, BLaER1 cells were adjusted to a density of 3.3×10^5^ cells/ml in BLaER1 transdifferentiation medium (Tables S4, S5). Three milliliters of the cell suspension were seeded per well in 6-well plates (TPP) and incubated at 37°C, 5% CO_2_ for 7 days. Every 2-3 days, 1.5 ml of medium was replaced with fresh transdifferentiation medium.

After 7 days, undifferentiated suspension cells were removed by aspiration of the supernatant. The transdifferentiated, adherent cells were subsequently harvested by thorough washing of the wells with medium. The BLaER1 cells were resuspended at a density of 4×10^5^ cells/ml in transdifferentiation medium. Of this suspension, 5 ml were seeded in 6-well plates (TPP), 200 μl per well were seeded into 96-well plates (TPP), 300 μl in 96-well square glass-bottom plates (ibidi), 1 ml in 24-well plates with a coverslip (TPP) for immunofluorescence staining, 250 μl in 8-well slides (ibidi) or 300 μl in 12-well removable chamber slides (ibidi). Cells were allowed to attach at room temperature (RT) for 20-30 min and were then incubated at 37°C, 5% CO_2_ overnight prior to experimentation.

Cells were monitored microscopically at each medium exchange. Transdifferentiated cells were identified by GFP positivity, adherence, and an elongated, spindle-shaped, macrophage-like morphology. Cell lines were regularly checked for the absence of *Mycoplasma* contamination. The identity of all cell lines was regularly confirmed by STR analysis.

### Monocyte isolation and differentiation

Human peripheral blood mononuclear cells were isolated from buffy coats obtained from healthy donors by density gradient centrifugation using Histopaque-1077 (Sigma-Aldrich). CD14^+^ monocytes were subsequently purified by magnetic-activated cell sorting (MACS®) (Table S5) using the autoMACS® system (Miltenyi Biotec), in accordance with the manufacturer's instructions.

Purified CD14^+^ monocytes were resuspended in hMDM differentiation medium (Table S4) and seeded at 1×10^7^ cells into 175 cm^2^ tissue culture flasks (Sarstedt). Cells were differentiated for 7 days at 37°C and 5% CO_2_. Medium was exchanged after 5 days.

Following differentiation, macrophages (Table S2) were detached using 50 mM EDTA in PBS and resuspended at a density of 2×10^5^ cells/ml. For subsequent experiments, seeding was performed as described above for the BLaER1 cells.

### Cultivation of fungi

All fungi used in this study were routinely cultured in yeast extract peptone dextrose (YPD) medium (Table S4). All *C. albicans* strains (Table S3), as well as *C. auris* (Table S3), were grown under shaking conditions at 30°C and 180 rpm for 16 h prior to infection. *C. glabrata* (Table S3) was cultured at 37°C, 180 rpm shaking for the same duration. Following growth, fungal cultures were washed with PBS, counted using Neubauer hemocytometers, and adjusted to an MOI of 3 (unless stated otherwise) in RPMI 1640 medium (Thermo Fisher Scientific).

### LPS priming of host cells

For RNA-sequencing and inflammasome activation studies, host cells were primed with 50 ng/ml LPS (Sigma-Aldrich) (Table S5) for 2 h at 37°C, 5% CO_2_ prior to infection. Afterwards, LPS-containing supernatant was aspirated and fresh RPMI 1640 medium was added.

### Opsonization of fungi

Human blood was drawn from healthy volunteers and collected in an S-Monovette® Serum CAT (Sarstedt) after informed written consent. Serum was obtained after blood components were separated by centrifugation (2000 ***g***, 10 min) and the serum (upper phase) was harvested or from pooled human blood serum (Bio&SELL). For the respective experiments, fungal overnight cultures were harvested as described before and pelleted by centrifugation (10,000 ***g***, 30 s). The cell pellet was resuspended in blood serum (100 μl per 2×10^7^ cells) and opsonized for 1 h on ice. Opsonized fungi were pelleted by centrifugation (10,000 ***g*** for 30 s), resuspended in RPMI 1640 medium, and diluted to the desired concentration for the infection.

### Infection of host cells and synthetic candidalysin treatment

One day after seeding, the medium from BLaER1 cells and hMDMs was removed and, if required, LPS priming was performed as described above. In parallel, preparation of fungal overnight cultures and, optionally, opsonization were performed as previously described. Afterwards, fresh, pre-warmed RPMI 1640 (unless stated otherwise) was added to half of the final volume (100 μl for 96-well plates, 150 μl in 96-well square glass-bottom plates, 500 μl for 24-well plates, 125 μl for 8-well slides, 150 μl for 12-well removable chamber slides). Finally, the corresponding volume of fungal suspension adjusted to MOI 3 (unless stated otherwise) was added to the cells and incubated at 37°C, 5% CO_2_ for the desired time. Synthetic candidalysin (Table S5) used to treat the host cells was custom synthesized (Peptide Synthetics) and dissolved in cell culture-grade DMSO to a concentration of 10 mM. Before treatment, candidalysin was pre-diluted to 40 μM in RPMI and finally added to the host cells at a ratio of 1:2 (final concentration: 20 μM).

### Flow cytometry

To validate the transdifferentiation of BLaER1 cells, expression of the immune cell surface markers CD11b (ITGAM), CD14 and CD19 was analyzed by flow cytometry (Table S5). A total of 6×10^5^ undifferentiated or transdifferentiated BLaER1 cells were collected in 96-well V-bottom plates (Greiner) by centrifugation for 4 min at 340 ***g*** and 4°C. Cells were blocked for 15 min on ice using 2.5 mM EDTA/2% FBS, (FACS sample buffer)+10% human serum in PBS, washed once with 2.5 mM EDTA/2% FBS in PBS, and stained for 60 min with fluorochrome-conjugated antibodies (Table S5) CD11b–PE/Cyanine7 (1:66.67, BioLegend), CD14–Pacific Blue (1:333.33, BioLegend) and CD19–PerCP/Cy5.5 (1:66.67, BioLegend) diluted in FACS sample buffer.

To assess the expression of macrophage polarization markers (M1/M2) and PRRs, hMDMs and BLaER1 cells were seeded in independent experiments and optionally LPS-primed. Afterwards, cells were collected and incubated with Fc Receptor Blocking Solution (1:50; BioLegend) to prevent nonspecific antibody binding. The M1/M2 panel (Table S5) included CD163–APC (1:50; BioLegend), CD80–PE (1:50; BioLegend), CD206–BV421 (1:50; BioLegend), CD11c–APC-Cy7 (1:50; BioLegend) and CD14–PerCP-Cy5.5 (1:100; BioLegend). The PRR panel consisted of Dectin-3–PE (1:50; Miltenyi Biotec), TLR4–BV421 (1:50; BioLegend), Dectin-1–APC (1:50; BioLegend), TLR2–PE–Cy7 (1:50; BioLegend) and CD14–PerCP-Cy5.5 (1:50; BioLegend) all diluted in FACS sample buffer.

Viability of BLaER1 cells was assessed based on GFP expression, with GFP^+^ cells considered viable and GFP^−^ cells considered non-viable. Non-viable hMDMs were identified using 0.1 μM SYTOX Green (Table S5). Samples were acquired on a FACSVerse Cell Analyzer (Becton, Dickinson and Company) and analyzed using FlowJo software (Becton, Dickinson and Company).

### RNA isolation and sequencing

Host cells were seeded in 6-well plates (TPP) and primed with LPS as described above. Following priming, LPS-containing medium was removed. Subsequently, the cells were left uninfected or infected with *C. albicans* SC5314 at an MOI of 3 for 2 h. After incubation, RNA was isolated using the RNeasy Mini kit (QIAGEN) according to the manufacturer's instructions. Prior to sequencing, RNA concentrations were quantified using a NanoDrop 1000 Spectrophotometer (Thermo Fisher Scientific) and RNA quality was determined using a BioAnalyzer 2100 (Agilent Technologies) (Table S5). RNA sequencing was carried out at Novogene (Munich, Germany) with 2×150 bp paired reads using an Illumina NovaSeq X Plus instrument. Sample reads were trimmed with Trimmomatic (v0.39) (Bolger et al., 2014) to remove possible Illumina adapters, then aligned with HISAT2 (v2.2.1) (Kim et al., 2019) to the human genome version GRCh38.p14. Reads per gene were determined using featureCounts of the SubRead package (v2.0.3) (Liao et al., 2014) and the GENCODE primary assembly annotation (v49) (Mudge et al., 2025), using the ‘fraction’ option for multi-overlapping reads counting (-O option). The count data were evaluated using the DESeq2 package (v1.50.2) (Love et al., 2014) in R (v4.5.1) (http://www.r-project.org/) with the design based on cell type, priming, and infection state, filtered on a minimum of ten reads per feature. For the correlation of infection-responsive genes, the subset of LPS-primed, infected hMDMs were compared to LPS-primed, uninfected hMDMs, with a donor-aware design to compensate for individual expression differences. Features with significant upregulation (*P*<0.05, log2FC>1) were selected from the hMDMs contrast ‘infected versus uninfected’, their log2FC plotted against the log2FC of the BLaER1 cells data subset, and the Spearman correlation coefficient determined by the R cor.test function.

### Phagocytosis and hyphal length assays

Prior to infection, *C. glabrata* and *C. auris* were stained with RBITC. To this end, overnight cultures were prepared as described above, washed twice in PBS and resuspended in 0.1 M sodium bicarbonate buffer (pH 9.0). RBITC was added to a final concentration of 20 μg/ml. After an incubation step at RT with shaking for 30 min, the fungal solution was washed twice with PBS, resuspended in PBS, and adjusted to an MOI of 1 and 3.

Host cells were seeded in separate 8-well slides for each fungus and timepoint and infected with SC5314-mScarlet or RBITC-stained *C. glabrata*/*C. auris* in RPMI 1640 medium without Phenol Red (Thermo Fisher Scientific) to reduce background fluorescence. After addition of the fungus, the slides were incubated for 0.5, 1, 3 and 5 h at 37°C 5% CO_2_. In parallel, 3.5 mg/ml CFW (Sigma-Aldrich) (Table S5) was sterile-filtered, diluted 1:11.6667 in sterile, deionized water and kept on ice. Similarly, 5 mg/ml ConA-AF350 (Thermo Fisher Scientific) (Table S5) was diluted 1:20 in deionized water and kept at 4°C. At the desired timepoints, the infected slides were removed from the incubator, the pre-diluted CFW solution was added to the *C. albicans*- and *C. auris*-infected samples at a 1:100 ratio and left to bind for 10 min at RT. Similarly, for *C. glabrata*-infected samples, the pre-diluted ConA-AF350 solution was added at a 1:100 ratio and incubated for 10 min at RT.

Afterwards, the slides were imaged using an inverted Axio Observer.Z1 microscope (Zeiss) with a 40×/1.1 numerical aperture water immersion objective (Zeiss) and the following filters: brightfield, no filter; CFW/Alexa Fluor 350, excitation 385±15 nm and emission 425±13 nm; GFP, excitation 464±14 nm and emission 525±25 nm; mScarlet/RBITC, excitation 555±15 nm and emission 605±35 nm. At least four different, randomly selected positions across two technical replicates were imaged.

Afterwards, images were analyzed in ZEN 3.10 software (Zeiss) using the same display settings for each condition. The number of total fungi per field of view were counted, using an overlay of brightfield and mScarlet channel for *C. albicans*, brightfield, Alexa Fluor 350 and RBITC channel for *C. glabrata* and brightfield, CFW and RBITC for *C. auris*. Finally, the number of phagocytosed fungi was determined by counting CFW^−^ *C. albicans* or *C. auris* and Alexa Fluor 350^−^ *C. glabrata*. On average, 493 fungal cells were counted per replicate. The phagocytosis ratio was calculated as the average number of phagocytosed fungi divided by the number of total fungi per condition. Additionally, hyphal length of at least 40, fully intracellular hyphae (CFW^−^) was measured using the spline curve tool and the average hyphal length per condition was calculated.

### Phagolysosome acidification assay

Host cells were seeded in 8-well slides. Prior to infection, SC5314 cells were heat-killed at 70°C for 10 min and included as a positive control for phagolysosome acidification. Afterwards, live and heat-killed SC5314 as well as *cph1*Δ/Δ*efg1*Δ/Δ cells were adjusted to an MOI of 1 as described above, and host cells were infected. The acidophilic dye LysoTracker Red DND-99 (Thermo Fisher Scientific) (Table S5) was added to a final concentration of 75 nM and the infected cells were immediately imaged live in the Celldiscoverer 7 imaging system (Zeiss) with the 50×/1.2 numerical aperture water immersion objective (Zeiss) and 1× tube lens under incubation (37°C, 5% CO_2_). Images of four adjacent positions with a 10% overlap were acquired for 6 h, at a 30 min interval with the following filters: brightfield, no filter; GFP, excitation 425±13 nm and emission 524±23 nm; LysoTracker Red DND-99, excitation 555±15 nm and emission 594±9 nm. Images were evaluated using ZEN 3.10 software. The percentage of LysoTracker^+^ phagolysosomes was calculated as the ratio of LysoTracker^+^ foci and the total number of phagocytosed fungi per field of view.

### Immunofluorescence of *Candida*-containing phagolysosomes

Host cells were seeded in 12-well removable chamber slides. SC5314 was heat-killed as described above and the host cells were infected with heat-killed SC5314, as well as live SC5314, and *cph1*Δ/Δ*efg1*Δ/Δ strains. Subsequently, phagocytosis was synchronized by resting the slides on ice for 20 min. Afterwards, the slides were incubated for 1 h at 37°C, 5% CO_2_. Alternatively, host cells were primed with LPS as described above and infected with SC5314, *cph1*Δ/Δ*efg1*Δ/Δ, *cph1*Δ/Δ*efg1*Δ/Δ-candidalysin overexpression or *eed1*Δ/Δ strains. Subsequently, the slides were incubated for 3 h at 37°C, 5% CO_2_. Following incubation, the medium was aspirated, and slides were fixed with 4% Histofix (Roth) for 15 min at 37°C, 5% CO_2_ and washed three times with PBS. Immunofluorescence staining was performed by blocking the slides with 5% bovine serum albumin (BSA)+5% goat serum for 1 h at RT. After blocking, cells were permeabilized with 0.5% Triton X-100 (Sigma-Aldrich) for 5 min at RT and subsequently washed three times with PBS. Afterwards, total fungal cells were stained with CFW diluted in deionized water at a ratio of 1:100 for 20 min in the dark. Following incubation with CFW, the slides were washed with deionized water three times for 5 min at 30°C. Samples were then stained in parallel with mouse anti-LAMP1 antibody (sc-20011, Santa Cruz Biotechnology) and/or rabbit anti-RAB7 antibody (9367, Cell Signaling Technology) (Table S5) diluted at a ratio of 1:100 in 2% BSA overnight at 4°C in the dark. For hMDMs, the actin cytoskeleton was additionally labeled with phalloidin–Alexa Fluor 750 (Table S5) diluted at a ratio of 1:400 in 1% BSA for 45 min at RT in the dark. On the following day, samples were washed three times with PBS and secondary antibodies anti-mouse-Alexa Fluor 647 (A32728, Thermo Fisher Scientific) and anti-rabbit-Alexa Fluor 568 (A-11011, Thermo Fisher Scientific) (Table S5) diluted at a ratio of 1:250 in 2% BSA were added for 1 h at RT in the dark. Afterwards, samples were washed three times with PBS.

Following staining, samples were mounted with ProLong Diamond Mounting Medium (Thermo Fisher Scientific) and left in the dark at RT to dry overnight. Samples were imaged in the inverted Axio Observer.Z1 microscope (Zeiss) using a 40×/1.1 numerical aperture water immersion objective (Zeiss) and the following filters: brightfield, no filter; CFW, excitation 385±15 nm and emission 425±13 nm; GFP/Alexa Fluor 488, excitation 464±14 nm and emission 525±25 nm; Alexa Fluor 568, excitation 555±15 nm and emission 605±35 nm; Alexa Fluor 647, excitation 631.5 nm±16.5 nm and emission 681±19 nm; Alexa Fluor 750, excitation 735 nm±15 nm and emission 785±15 nm. At least three different, randomly selected positions per condition were imaged. For visualization of the full hyphae at the 5 h timepoint, *z*-stacks capturing the entire hypha were acquired using the *z*-stack function of the software (ZEN v3.10) with optimal slice spacing.

Image analysis was performed using ZEN software v3.10. For the 5 h images, maximum-intensity projection was performed and background was subtracted using the ‘Rolling Ball’ tool (radius: 100). Subsequently, for all channels and images, display settings were adjusted using the automatic minimum/maximum scaling function and an overlay of the GFP and CFW channels was used to identify phagolysosomes containing fungal cells.

The method of quantification was based on Kasper et al. (2018). Per field of view, approximately 10-15 events were selected for analysis. On average, 76 phagolysosomes were analyzed per condition. Recruitment of endosomal markers was assessed using the respective single-channel images. Phagolysosomes were scored as marker-positive when a clear accumulation of fluorescence signal was observed surrounding the fungal cell. Positive staining was defined by a signal that followed the shape of the fungal cell and displayed a higher intensity than the average cytoplasmic background of the host cell. The number of RAB7^+^ and LAMP1^+^ phagolysosomes was determined and expressed as a percentage of the total number of analyzed events per condition.

### Host cell death analysis

Kinetics of macrophage cell death were assessed by time-lapse fluorescence microscopy. hMDMs and BLaER1 cells were seeded into 96-well, square glass-bottom plates (ibidi). If required, cells were LPS-primed as described above. Cells were then infected with the corresponding *C. albicans* strains at MOI 1 for BLaER1 cells and MOI 3 for hMDMs or co-incubated with synthetic candidalysin. For hMDMs, all cells were labeled with 1 μM SYTO Deep Red (Table S5), and non-viable cells were detected with 0.1 μM SYTOX Green, whereas BLaER1 cell numbers were determined based on constitutive GFP expression. Plates were transferred to the Celldiscoverer 7 (Zeiss) and imaged with a 20×/0.7 numerical aperture objective (Zeiss) and 1× tube lens under incubation (37°C, 5% CO_2_). Images of four adjacent positions with a 10% overlap were acquired for 24 h, at a 30 min interval with the following filters: brightfield, no filter; GFP/SYTOX Green, excitation 425±13 nm and emission 524±23 nm; SYTO Deep Red, excitation 555±15 nm and emission 594±9 nm.

Background was subtracted in ZEN 3.10 (Zeiss) using the Rolling Ball tool (size: 100) and exported as .jpg. Afterwards, image analysis was performed using Fiji (ImageJ). SYTO Deep Red and GFP/SYTOX Green images were initially converted to 8-bit and subsequently to binary format using the threshold function (settings: 20, 255) and particles separated using the watershed function. Finally, positive cells were quantified using the ‘Particle Analyzer’ tool (size: 15-infinity; circularity: 0.04-1.00). The total number of hMDMs was determined from SYTO Deep Red^+^ cells in the first acquired frame, which served as the normalization reference for subsequent timepoints. For BLaER1 cells, total cell numbers were determined based on all GFP^+^ cells in the first timepoint, and subsequent loss of GFP signal was interpreted as cell death.

### Quantification of cytokine release

BLaER1 cells were seeded in 96-well plates and infected with the respective *C. albicans* strains. Nigericin (Sigma-Aldrich) (Table S5) was added as a positive control for IL-1β release (final concentration: 5 μM). The cells were incubated for 5 h or 24 h (37°C, 5% CO_2_) and centrifuged (250 ***g***, 10 min). The supernatants were transferred into a fresh 96-well plate, sealed with a film, and stored at −20°C. Prior to the cytokine measurements, the plates were thawed overnight at 4°C and centrifuged to remove debris (250 ***g***, 10 min). Samples were adequately diluted in 1% biotin-free BSA (Roth) in PBS (see Table S6) and cytokine concentrations were determined with an ELISA assay using DuoSet ELISA kits (R&D Systems) (Table S5) following the manufacturer's protocol. All samples were measured in technical duplicate or triplicate (measurement at 450 nm; reference at 570 nm) with an Infinite M200 Pro plate reader (Tecan). To calculate cytokine concentrations, the mean blank absorbance value was subtracted from all samples and cytokine standards and the concentrations were calculated according to the standard curve by applying a linear (IL-1β, IL-6 and TNFα) or a second-degree polynomial (IL-8) regression. Values below the lowest standard concentration were considered as 0 pg/ml.

### Quantification of host cell damage

Lytic damage to BLaER1 and hMDMs was assessed by quantifying the release of cytosolic LDH into the medium after infection with *C. albicans* for 5 and 24 h. Following infection, the plates were centrifuged at 250 ***g*** for 10 min, the supernatants were collected and diluted at a ratio of 1:5 in PBS. LDH concentrations were measured using the Cytotoxicity Detection kit (Roche) (Table S5) according to manufacturer's instructions.

### ASC speck formation

BLaER1 WT, *GSDMD^−/−^* and hMDMs cells were seeded into 12-well, removable chamber slides (Ibidi). Cells were primed with LPS and subsequently infected with *C. albicans* at an MOI of 3. As a positive control for inflammasome activation, cells were stimulated with nigericin (10 μg/ml) for 30 min.

At 2 h p.i., samples were fixed and stained as described above with rabbit anti-ASC antibody (1:100; 67824, Cell Signaling Technology) and anti-rabbit Alexa Fluor 568 secondary antibody (1:500; A-11011, Thermo Fisher Scientific) (Table S5).

Fungal structures were visualized using CFW (Sigma-Aldrich) as described above. For hMDMs, the actin cytoskeleton was additionally labeled with phalloidin–Alexa Fluor 488 (Table S5) diluted at a ratio of 1:400 in 1% BSA for 45 min at RT in the dark. Slides were mounted, and fluorescence images were acquired as described above.

Afterwards, display settings were linearly adjusted to minimum/maximum and background was subtracted using the ‘Rolling Ball’ tool (radius: 100) in ZEN 3.10 (Zeiss). Subsequently, ASC specks were identified in infected cells, in which cytosolic ASC staining had concentrated into a single, bright fluorescent spot.

### Statistical analysis

Experiments were performed with at least three biological replicates. For experiments with hMDMs, with at least three different donors on two different days. Data were analyzed using GraphPad Prism 10.5.0 (GraphPad Software).

### Use of artificial intelligence

ChatGPT (GPT-5.2) was used to assist in drafting portions of the Materials and Methods section. All content was reviewed, edited and approved by the authors, who take full responsibility for its accuracy.

## Supplementary Material

10.1242/dmm.052865_sup1 Supplementary information

Table S1. Differentially-expressed genes between LPS-primed BLaER1 cells and hMDMs Gene expression of uninfected LPS-primed BLaER1 cells vs. LPS-primed hMDMs was analyzed by RNA sequencing. Log2-transformed fold change (log2FC) of differentially expressed genes (p adjusted<0.05) either upregulated in BLaER1 cells (log2FC>1) or upregulated in hMDMs (log2FC<1)
