# Supplementary material for: Transdifferentiated BLaER1 cells as a genetically tractable model to study the interaction of pathogenic fungi with macrophages
Source: Dis Model Mech. 2026 Aug 5;19(8):dmm052865. doi: 10.1242/dmm.052865 (PMC13488413; doi:10.1242/dmm.052865)
Supplement: Supplementary file 1 [file dmm-19-052865-s1.pdf]

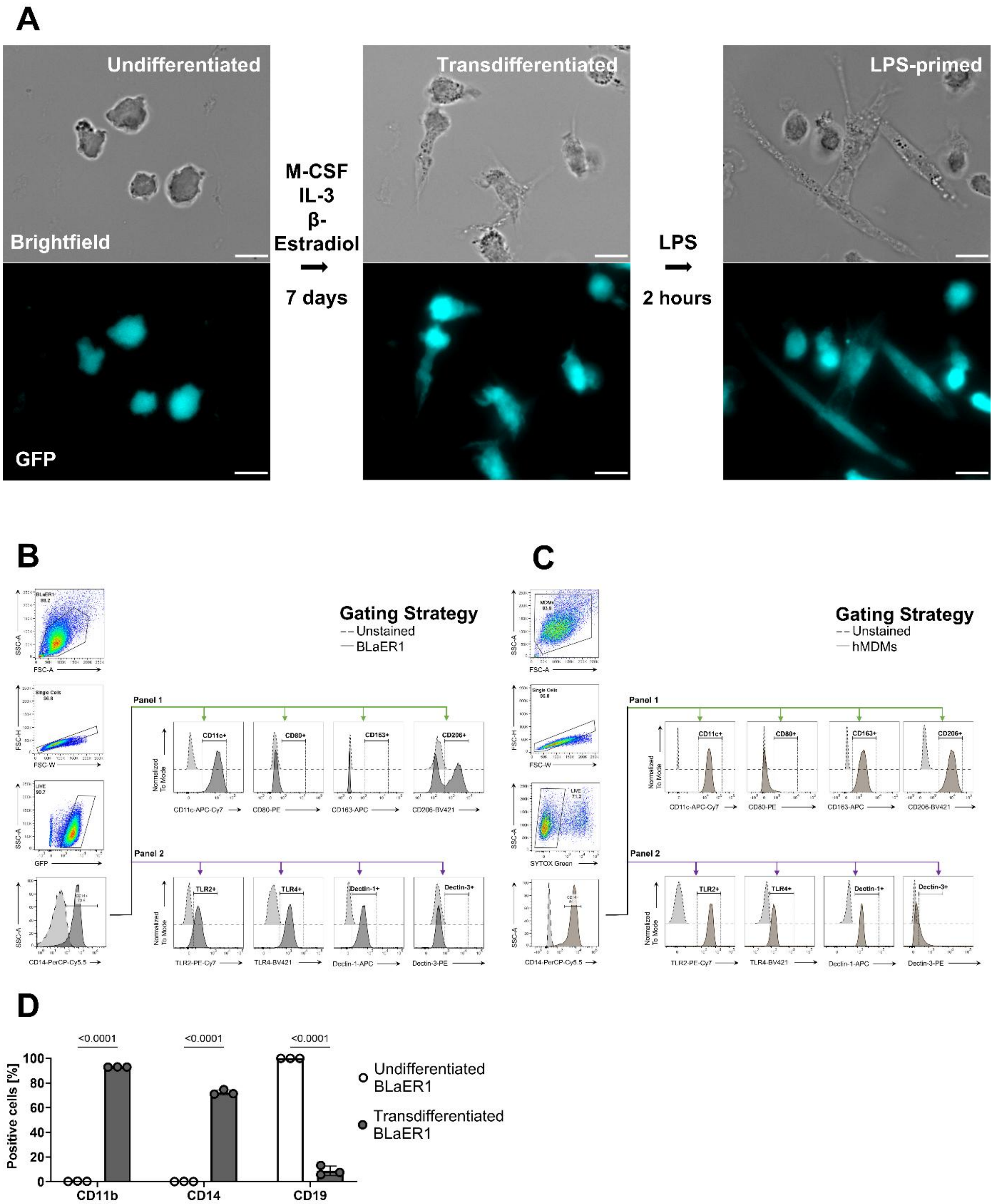

Fig. S1. BLaER1 cell transdifferentiation is efficient

(A) Morphological changes of BLaER1 cells upon transdifferentiation and LPS priming were tracked microscopically. Representative regions, 630× magnification, contrast was linearly adjusted to minimum

Disease Models & Mechanisms • Supplementary information

and maximum values, scale bar = 50 µm. (B) Gating strategies for the flow cytometry analysis of BLaER1 cells. Single, living cells were identified by FSC, SSC, and cytosolic GFP expression. Marker positive cells were identified in comparison to unstained controls. (C) Gating strategies for the flow cytometry analysis of hMDMs. Gating was performed similar to the BLaER1 cells. SYTOX Green was used as a viability marker instead of cytosolic GFP. (D) Transdifferentiation efficiency of BLaER1 cells was quantified by flow cytometry. The number of positive cells was determined as percentage of GFP^+ cells ($n = 3$). Data is displayed as mean ± s.d. and was analyzed with a one-way ANOVA and Tukey's multiple comparisons test. Only p-values below 0.05 are indicated.

Disease Models & Mechanisms • Supplementary information

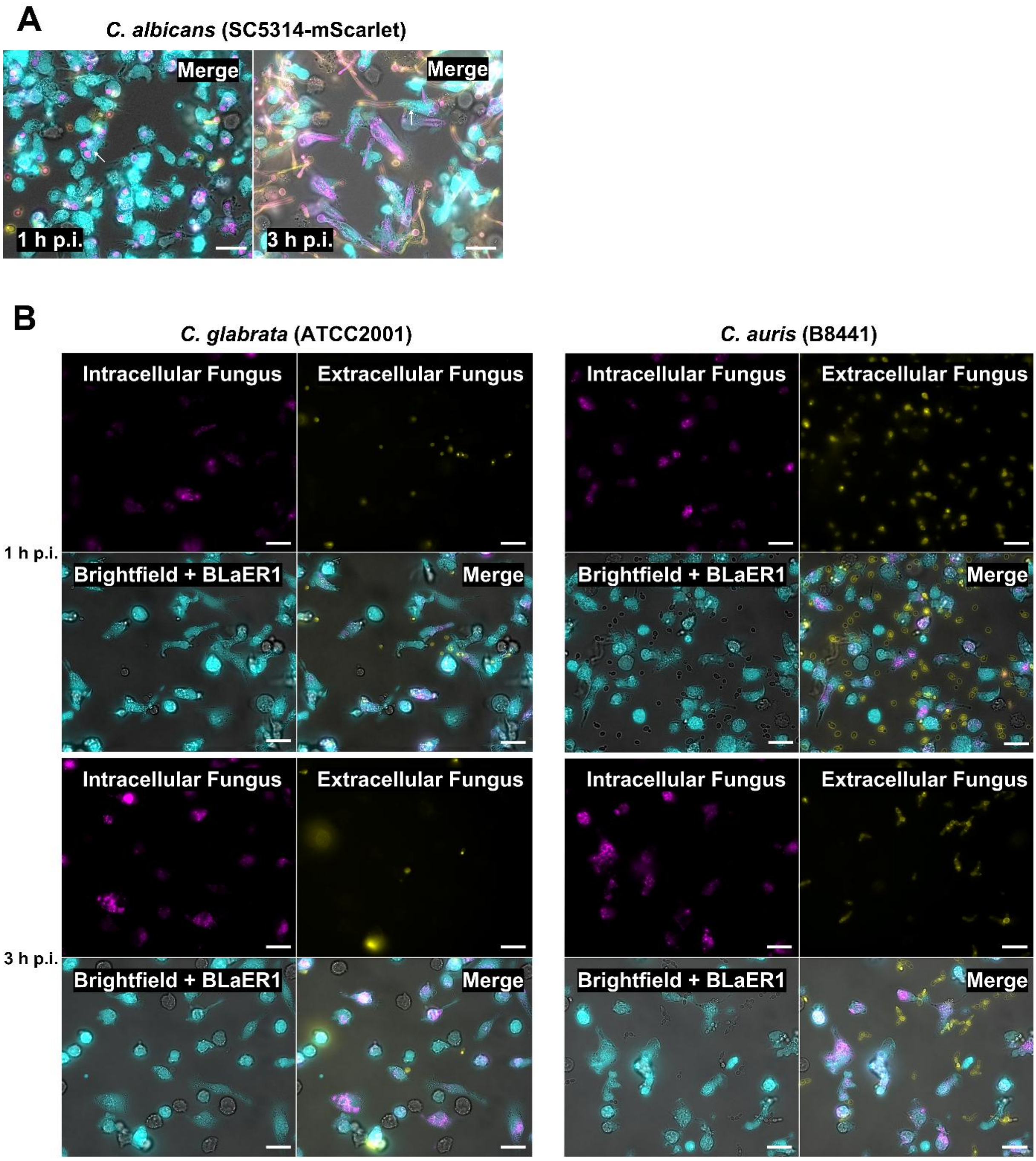

Fig. S2. Phagocytosis of *C. albicans, C. glabrata,* and *C. auris* by BLaER1 cells progresses after 30 min (A, B) Phagocytosis of *C. albicans* SC5314-mScarlet WT (A), RBITC-labeled *C. glabrata* ATCC2001 WT (B, left panel) and *C. auris* B8441 WT (B, right panel) by BLaER1 cells was visualized by live-cell imaging. Extracellular fungal cells were stained with calcofluor white (*C. albicans* and *C. auris*) or concanavalin A-Alexa Fluor 350 (*C. glabrata*) at 1 h and 3 h p.i. Representative regions, 400x magnification, contrast was linearly adjusted to minimum and maximum values, scale bar = 20 µm.

Disease Models & Mechanisms • Supplementary information

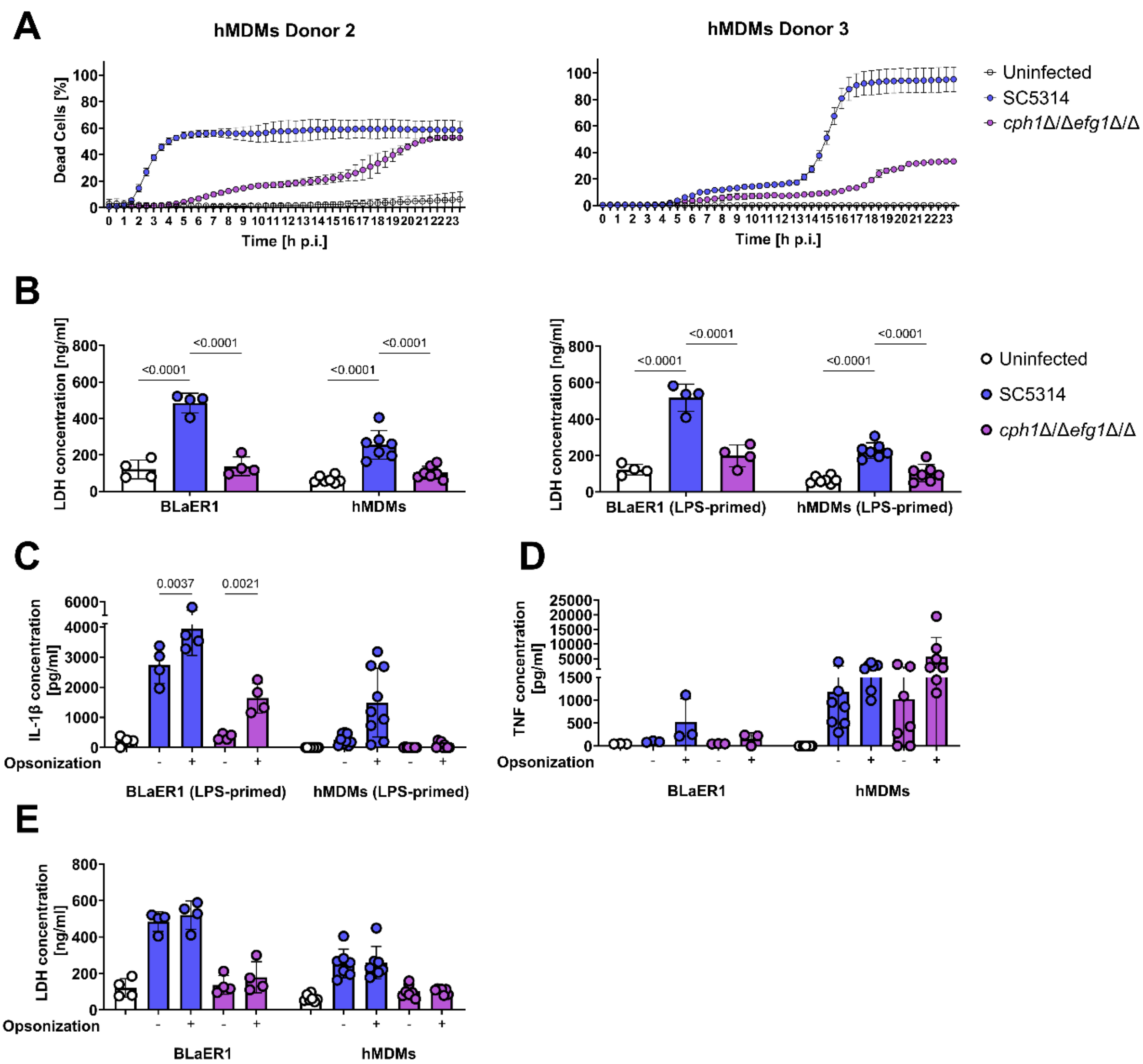

Fig. S3. *C. albicans* infection of BLaER1 cells and hMDMs leads to filamentation-dependent cell death, damage, and pro-inflammatory immune responses in the host, which can be enhanced by opsonization of the fungus (A) Cell death of hMDMs uninfected, infected with *C. albicans* SC5314 WT or the non-filamentous *cph1*Δ/Δ*efg1*Δ/Δ mutant was tracked *via* live-cell imaging in presence of the membrane impermeable dye SYTOX Green. Total cell number was determined based on SYTO Deep Red$^+$ nuclei and normalized to the first time point (one donor per graph, error bars represent three technical replicates). (B) LDH release from unprimed and LPS-primed BLaER1 cells and hMDMs was quantified 24 h p.i. with the same strains as in (A) (BLaER1: n = 4, hMDMs: 7 donors). (C) Secretion of IL-1β from LPS-primed BLaER1 cells and LPS-primed hMDMs was quantified 5 h p.i. with unopsonized or serum-opsonized *C. albicans* SC5314 WT or a non-filamentous *cph1*Δ/Δ*efg1*Δ/Δ mutant strain (BLaER1: n = 4, hMDMs: 9 donors). (D, E) Secretion of TNF (D) or release of LDH (E) from unprimed BLaER1 cells and hMDMs was quantified 24 h p.i. with the same strains as in (C). Data points of the unopsonized strains are the same as in Fig. 5 (IL-1β, TNF) and (B, this figure) (BLaER1: n = 3 and 4, hMDMs: 7 donors). Values of 0 pg/ml represent 'not detected' Data is displayed as mean ± s.d. and was analyzed with a two-way ANOVA and Tukey's multiple comparisons test. Only p-values below 0.05 are indicated.

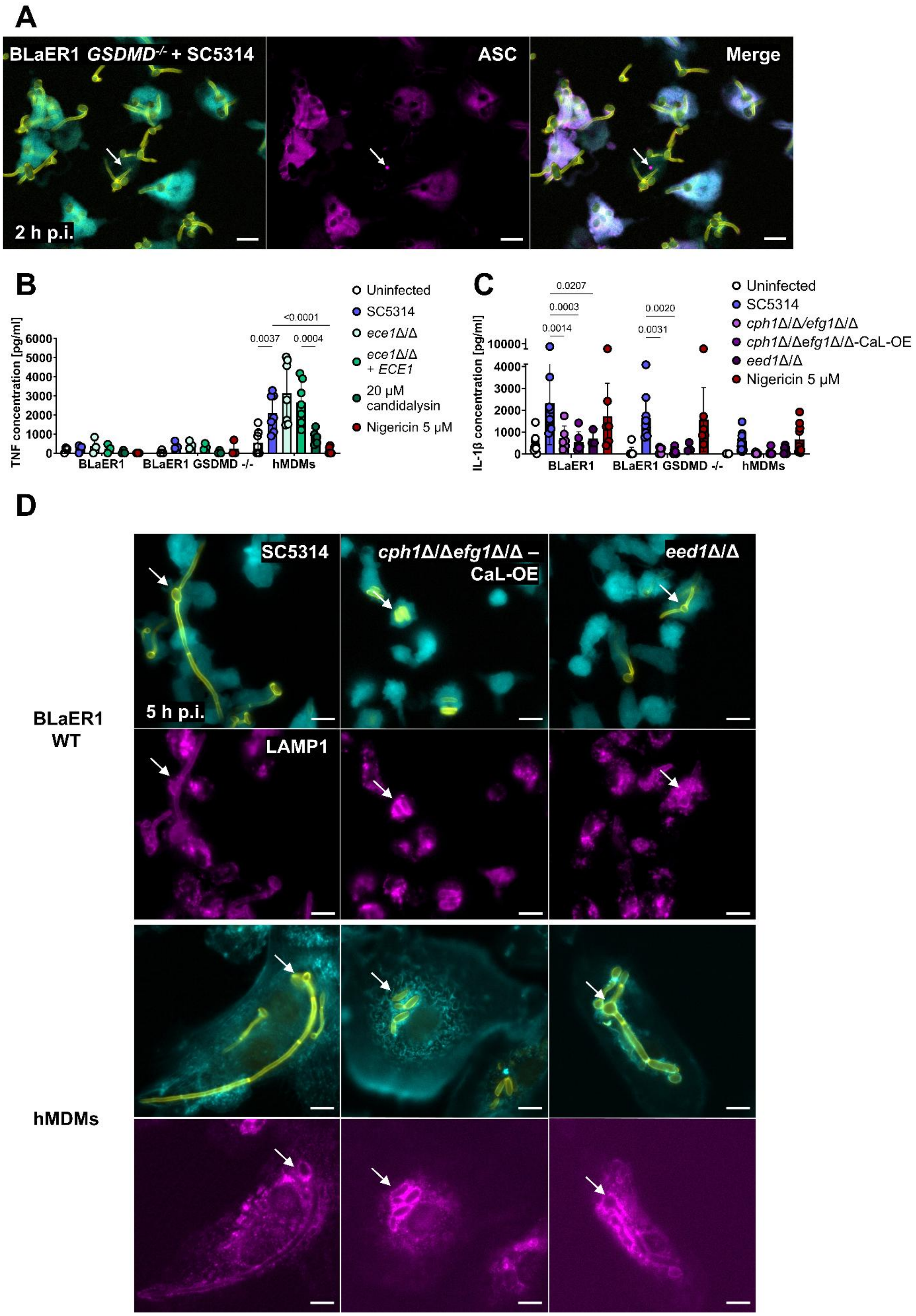

Disease Models & Mechanisms • Supplementary information

Fig. S4. Candidalysin-induced inflammasome activation requires C. albicans filamentation. (A) ASC speck formation (stained with Alexa Fluor 647) in LPS-primed BLaER1 *GSDMD*$^{-/-}$ cells was visualized by immunofluorescence microscopy 2 h p.i. with *C. albicans* SC5314 WT. Total fungus was stained with calcofluor white. Representative region, 400x magnification, contrast was linearly adjusted to minimum and maximum values, scale bar = 10 µm. (B) Secretion of IL-1β from LPS-primed BLaER1 cells and LPS-primed hMDMs was quantified by ELISA 5 h p.i. with *C. albicans* SC5314 WT, non-filamentous *cph1*Δ/Δ*efg1*Δ/Δ, *cph1*Δ/Δ*efg1*Δ/Δ-candidalysin overexpression (*cph1*Δ/Δ*efg1*Δ/Δ-CaL-OE), and *eed1*Δ/Δ mutant strains or treated with 5 µM nigericin as positive control. Data points of the uninfected, SC5314 infection and nigericin treatment include data from Fig. 6 (BLaER1: n 3-10, hMDMs: 6-16 donors). (C) LPS-primed BLaER1 cells and LPS-primed hMDMs were infected with the same strains as in (A). *C. albicans*-containing phagolysosomes 5 h p.i. were visualized by immunofluorescence microscopy. White arrows indicate LAMP1^{+}-phagolysosomes (stained with Alexa Fluor 647). Total fungus was stained with calcofluor white. Cytoskeleton of hMDMs was stained with phalloidin-Alexa Fluor 750. Representative regions, 400x magnification, maximum intensity projection, contrast was linearly adjusted to minimum and maximum values, scale bar = 10 µm. (D) Secretion of TNF from LPS-primed BLaER1 cells and LPS-primed hMDMs was quantified by ELISA 24 h after infection with *C. albicans* SC5314 WT, *ece1*Δ/Δ or *ece1*Δ/Δ+*ECE1* mutant strains or treatment with 20 µM synthetic candidalysin or 5 µM nigericin as positive control (BLaER1: n = 3, hMDMs: 7 donors). Values of 0 pg/ml represent 'not detected'. Data is displayed as mean ± s.d. and was analyzed with a one-way ANOVA and Tukey's multiple comparisons test (A, B hMDMs) or two-way ANOVA and Tukey's multiple comparisons test and (A, B BLaER1 cells). Only p-values below 0.05 of relevant comparisons are indicated.

Table S1. Differentially-expressed genes between LPS-primed BLaER1 cells and hMDMs

Gene expression of uninfected LPS-primed BLaER1 cells vs. LPS-primed hMDMs was analyzed by RNA sequencing. Log2-transformed fold change (log2FC) of differentially expressed genes (p adjusted<0.05) either upregulated in BLaER1 cells (log2FC>1) or upregulated in hMDMs (log2FC<1)

Available for download at
https://journals.biologists.com/dmm/article-lookup/doi/10.1242/dmm.052865#supplementary-data

Disease Models & Mechanisms • Supplementary information

Table S2. Host cells used in this study

	Source
**BLaER1**	Rapino et al. (2013)
**BLaER1 *GSDMD*$^{-/-}$**	Volkmar et al. (2024)
**hMDMs**	n/a

Table S3. Fungal strains used in this study

Strain	Internal Number	Parental	Genotype	Source
**SC5314**	C55	n/a	Clinical isolate, reference strain	Gillum et al. (1984)
**SC5314-mScarlet**	M2586	SC5314	*ADH1/adh1*::(tetO-P_{OP4}-*mScarlet*, *SAT1*)	Valentine et al. (2025)
ece1Δ/Δ	M2974	SC5314	*ece1*::Δ/*ece1*::Δ *NEUT5L/neut5l::FRT*	Weerasinghe et al. (2025 preprint)
ece1Δ/Δ+ECE1	M3026	M2974	*ece1*::Δ/*ece1*::*ECE1*, *FRT NEUT5L/neut5l*::*FRT*	Weerasinghe et al. (2025 preprint)
cph1Δ/Δefg1Δ/Δ	M2188	SC5314	*cph1*::*FRT*/ *cph1*::*FRT efg1*::*FRT*/ *efg1*::*FRT*	Wartenberg et al. (2014)
***cph1Δ/Δefg1Δ/Δ* – candidalysin overexpression**	M2360	BWP17	*cph1*::*hisG*/ *cph1*::*hisG efg1*::*hisG*/ *efg1*::*hisG-URA3-hisG caSAT1-pENO1-ECE1/ECE1*	Westman et al. (2018)
eed1Δ/Δ	M1315	SC5314		(Zakikhany et al., 2007)
**ATCC2001**	C94	*C. glabrata* WT	Isogenic WT	
**B8441**	CC221	*C. auris* WT, Clade I	Isogenic WT	(Lockhart et al., 2017)

Disease Models & Mechanisms • Supplementary information

Table S4. Media used in this study

Medium	Composition
**Yeast extract peptone dextrose**	1% yeast extract<br>2% peptone<br>2% glucose
**BLaER1 medium**	RPMI 1640<br>10 mM 4-(2-hydroxyethyl)-1-piperazineethanesulfonic acid (HEPES)<br>100 U/ml penicillin<br>100 µg/ml streptomycin<br>10% fetal bovine serum
**BLaER1 transdifferentiation medium**	BLaER1 Medium<br>10 ng/ml recombinant human macrophage colony-stimulating factor<br>10 ng/ml recombinant human interleukin-3<br>200 nM β-estradiol
**hMDMs differentiation Medium**	RPMI 1640<br>10% fetal bovine serum<br>50 ng/ml recombinant human macrophage colony-stimulating factor

Disease Models & Mechanisms • Supplementary information

Table S5. Key reagents and antibodies used in this study

Antibody (Clone)/Reagent	Supplier + Catalog number
**β-estradiol**	Sigma-Aldrich, E2758
**Recombinant human interleukin-3**	Immunotools, #11340035
**Recombinant human macrophage-colony-stimulating factor**	Immunotools, #11343115
**Lipopolysaccharide from Escherichia coli O55:B5**	Sigma-Aldrich, L2880
**Nigericin**	Sigma-Aldrich, SML1779
**Synthetic Candidalysin**	Peptide Synthetics, custom synthesis, Sequence: SIIGIIMGILGNIPQVIQIIMSIVKAFKGNK
**Anti-ASC/TMS1 (F8U7P)**	Cell Signaling Technology, 78000
**Anti-CD14 MACS beads**	Miltenyi Biotec, 130-050-201
**PE/Cyanine7 anti-CD11b (ICRF44)**	BioLegend, 301321
**APC-Cyanine7 anti-CD11c (Bu15)**	BioLegend, 337218
**Pacific Blue anti-CD14 (M5E2)**	BioLegend, 301816
**PerCP/Cyanine5.5 anti-CD14 (HCD14)**	BioLegend, 325622
**PerCP/Cyanine5.5 anti-CD19 (SJ25C1)**	BioLegend, 363016
**PE anti-CD80 (2D10)**	BioLegend, 305208
**APC anti-CD163 (RM3/1)**	BioLegend, 326510
**BV421 CD206 (15-2)**	BioLegend, 321126

Disease Models & Mechanisms • Supplementary information

**APC anti-Dectin-1 (15E2)**	BioLegend, 355405
**PE anti-Dectin-3 (REA757)**	Mitlenyi Biotec, 130-110-721
**PE/Cyanine7 anti-TLR2 (QA16A01)**	BioLegend, 153012
**BV421 anti-TLR4 (HTA125)**	BioLegend, 312811
**Calcofluor white (Fluorescent Brightener 28)**	Sigma-Aldrich, F3543
**Concanavalin A-Alexa Fluor 350**	Thermo Fisher Scientific, C11254
**LysoTracker Red DND-99**	Thermo Fisher Scientific, L7528
**Phalloidin-Alexa Fluor 488**	Thermo Fisher Scientific, A12379
**Anti-LAMP1 (H4A3)**	Santa Cruz Biotechnology, sc-20011
**Anti-RAB7 (D95F2)**	Cell Signaling Technology, 9367
**Anti-rabbit Alexa Fluor 568**	Thermo Fisher Scientific, A-11011
**Anti-mouse Alexa Fluor 647**	Thermo Fisher Scientific, A-21235
**SYTOX Green**	Thermo Fisher Scientific, S7020
**SYTO Deep Red**	Thermo Fisher Scientific, S34901
**Human TNF-alpha DuoSet ELISA**	R&D Systems, DY210
**Human IL-1 beta/IL-1F2 DuoSet ELISA**	R&D Systems, DY201
**Human IL-6 DuoSet ELISA**	R&D Systems, DY206

Disease Models & Mechanisms • Supplementary information

**Human IL-8/CXCL8 DuoSet ELISA**	R&D Systems, DY208
**Cytotoxicity Detection Kit (LDH)**	Sigma-Aldrich, 11644793001
**RNeasy Mini Kit**	Qiagen, 74104
**Agilent RNA 6000 Nano Kit**	Agilent Technologies, 5067-1511

Table S6. Dilution factors for cytokine measurements

Cytokine	Dilution hMDMs + hMDMs LPS-primed	Dilution BLaER1	Dilution BLaER1 LPS-primed
TNF	1:10	1:2	1:10
IL-1β	1:10	n.a.	1:20
IL-6	1:10	1:2	1:50
IL-8	1:100	1:50	1:100
